# Supplementary material for: Non-line-of-sight imaging with arbitrary illumination and detection pattern
Source: Nat Commun. 2023 Jun 3;14:3230. doi: 10.1038/s41467-023-38898-4 (PMC10239523; doi:10.1038/s41467-023-38898-4)
Supplement: Supplementary file 1 — Supplementary Information [file 41467_2023_38898_MOESM1_ESM.pdf]

Supplementary Information

Non-line-of-sight imaging with arbitrary illumination and detection pattern

Authors

Xintong Liu[1], Jianyu Wang[1], Leping Xiao[2,3], Zuoqiang Shi[1,4], Xing Fu[2,3] & Lingyun Qiu[1,4]

Affiliations

[1]Yau Mathematical Sciences Center, Tsinghua University, Beijing, 100084, PR China.
[2]State Key Laboratory of Precision Measurement Technology and Instruments, Department of Precision Instrument, Tsinghua University, Beijing, 100084, PR China.
[3]Key Laboratory of Photonic Control Technology (Tsinghua University), Ministry of Education, Beijing, 100084, PR China.
[4]Yanqi Lake Beijing Institute of Mathematical Sciences and Applications, Beijing, 101408, PR China.
✉email: fuxing@tsinghua.edu.cn; lyqiu@tsinghua.edu.cn

Contents

Supplementary Note 1: Additional experimental results
Reconstruction results of different experimental setups are shown.

Supplementary Note 2: The CC-SOCR algorithm
An iterative algorithm is designed to solve the proposed CC-SOCR optimization problem.

Supplementary Note 3: The choice of parameters
A self-adaptive scheme is used to determine the parameters in the CC-SOCR algorithm.

Supplementary Note 4: Time and memory complexity
A theoretical analysis of time and memory complexity is provided.

Supplementary Note 1 Additional experimental results

For all experiments, we interpolate the signals with the nearest neighbor method to bring F-K[1], LCT[2], D-LCT[3], PF-RSD[4] and SOCR[5] methods into comparisons unless otherwise stated. The coordinates of the focal points of all experiments are provided in the code.

Supplementary Figures 1 - 5 compare the reconstruction results of the bunny under different relay settings with the synthetic confocal signal provided in the Zaragoza dataset[6]. The illumination regions are shown in yellow. These results indicate the capability of the proposed CC-SOCR method in providing clear reconstructions of the hidden targets, even in cases with irregular relay settings.

Supplementary Figures 6 - 10 show the reconstruction results of the statue with the confocal measured signal provided in the Stanford dataset[1]. The exposure time of the original 512 × 512 confocal signal is 60 min. We sub-sample the signals with 10 × 10, 14 × 14, 18 × 18, 22 × 22, and 26 × 26 focal points in the area of 2 × 2 m^2. The reconstruction quality of all methods increases with the number of measurements. The CC-SOCR reconstructions are of high quality and contain very few background noise.

Supplementary Figures 11 - 17 compare the reconstruction results of the statue with different settings of the confocal measurements: 64 × 64 uniformly distributed focal points in an area of 2 × 2 m^2; a cross-shaped region that contains 1392 focal points; a set consisting of 200 randomly chosen focal points; a region consisting of 5 vertical bars with 1344 focal points; a region that consists of four letters N, L, O and S with 825 focal points; a region made up of several sticks sparsely and randomly distributed with 1229 focal points; and a heart-shaped region consisting of 258 focal points. For the case of a heart-shaped relay, the CC-SOCR method locates the target correctly, while all other methods fail.

Supplementary Figures 18 - 22 show the reconstruction results of the figure 4 with the measured non-confocal signal provided by the work of the phasor field method[4]. The detection point is fixed, which is 0.64 m to the left and 0.55 m to the bottom of the illumination region. We show reconstruction results under five different settings of the illumination regions: 64 × 64 uniformly distributed points in a square of size 1.27 × 1.27 m^2; five equispaced vertical bars that contain 3, 5, 5, 5, and 3 columns of illumination points from left to right; five equispaced horizontal bars that contain 3, 5, 5, 5, and 3 rows of illumination points from bottom to top; 14 × 14 uniformly distributed points; and 200 randomly distributed points. For the phasor field method, we show results of the PF-RSD method as it performs better than the PF-BP[7] method (See Fig. 7 in the main article). Both the SOCR and CC-SOCR methods provide noiseless reconstructions of the target in all scenarios, while the results of the CC-SOCR method contain fewer artifacts (Supplementary Figure 20) and are more accurate than the SOCR reconstructions (Supplementary Figures 21 - 22).

Supplementary Figure 23 shows the reconstruction results of the statue with different sizes of the virtual confocal signals introduced. The confocal signal is measured at 200 randomly distributed focal points in a square region of 2 × 2 m^2. The virtual confocal signals are also considered in this square region, and larger grid spacing leads to sparser virtual confocal signals. The reconstruction quality decreases with the size of the virtual confocal signal, which indicates the necessity of the dense virtual signal introduced. However, sparser virtual signals result in faster reconstruction processes (See Supplementary Tables 3 - 6), which shows the trade off between the reconstruction quality and the computation time.

Supplementary Figure 24 compares the F-K, LCT, D-LCT and SOCR reconstructions of the statue with the confocal signal measured at a heart-shaped region consisting of 258

focal points. The signal is preprocessed with zero padding and nearest neighbor interpolation techniques. It is shown that existing methods fail in this scenario.

Supplementary Figures 25 and 26 show reconstruction results of the statue with confocal signals measured at the letters N, L, O and S and a heart-shaped region. The least squares reconstructions without regularizations are of poor quality, while the CC-SOCR method reconstructs the targets faithfully.

Supplementary Figure 27 shows the reconstruction results of the statue with 10×10 confocal measurements using the F-K, LCT, D-LCT and SOCR methods. The signal is preprocessed with zero padding, nearest neighbor interpolation, linear interpolation, cubic interpolation and spline interpolation techniques. It is shown that the target cannot be clearly reconstructed with traditional signal interpolation techniques and conventional NLOS imaging algorithms due to the incompleteness of the measurements in spatial dimensions. The linear, cubic and spline interpolations result in C^0, C^1, and C^2 continuity, respectively. In general, the transient images with complete spatial measurements are not continuous due to the delta functions contained in the forward model (See equations (1) and (2) in the article). The nearest neighbor interpolation technique provides discontinuous results and is easy to implement for scenarios with arbitrary irregular measurement patterns. In all other experiments, the nearest neighbor interpolation technique is used.

Supplementary Figure 28 shows the reconstruction results of the letters N and T with non-planar and confocal measurements. The temporal resolution of the signal is 32 ps. The LOG-BP[8] and CC-SOCR algorithms directly work under irregular and non-planar relay settings. However, the LOG-BP method provides biased estimations of the targets with heavy background noise, while the proposed CC-SOCR method locates the targets correctly.

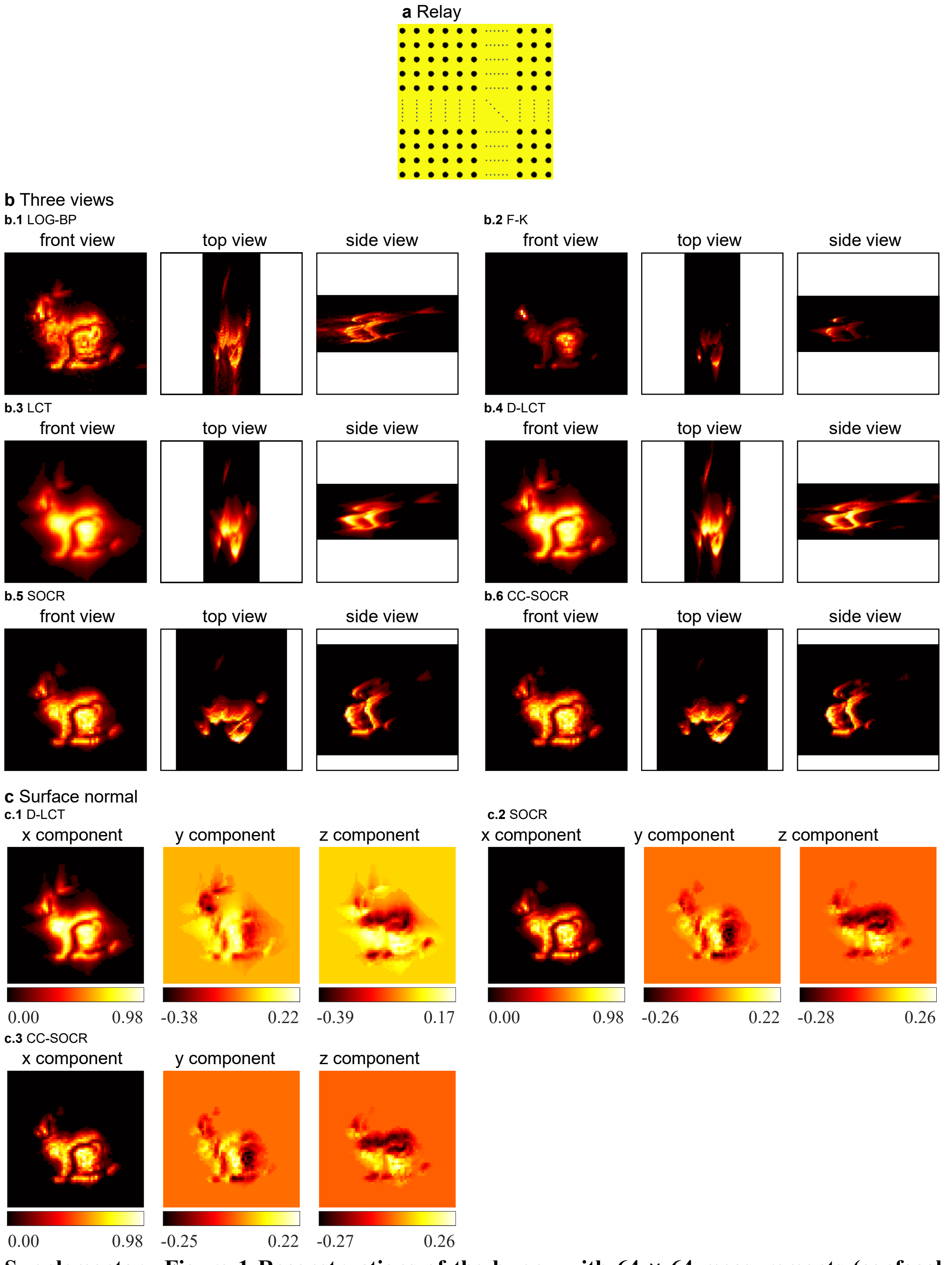

Supplementary Figure 1 Reconstructions of the bunny with 64 × 64 measurements (confocal, synthetic signal). a The relay is a square region. **b** Three views of the reconstructions. For all methods, the same reconstruction domain is shown. For the LOG-BP, F-K, LCT, and D-LCT methods, the length of the voxels in the depth direction is 0.125 cm. For the SOCR and CC-SOCR methods, the length of voxels in the depth direction is 0.25 cm. **c** The reconstructed surface normal of the D-LCT, SOCR and CC-SOCR methods is shown in the form of three components. The x, y and z components show values of the directional albedo in the depth, horizontal and vertical directions, respectively.

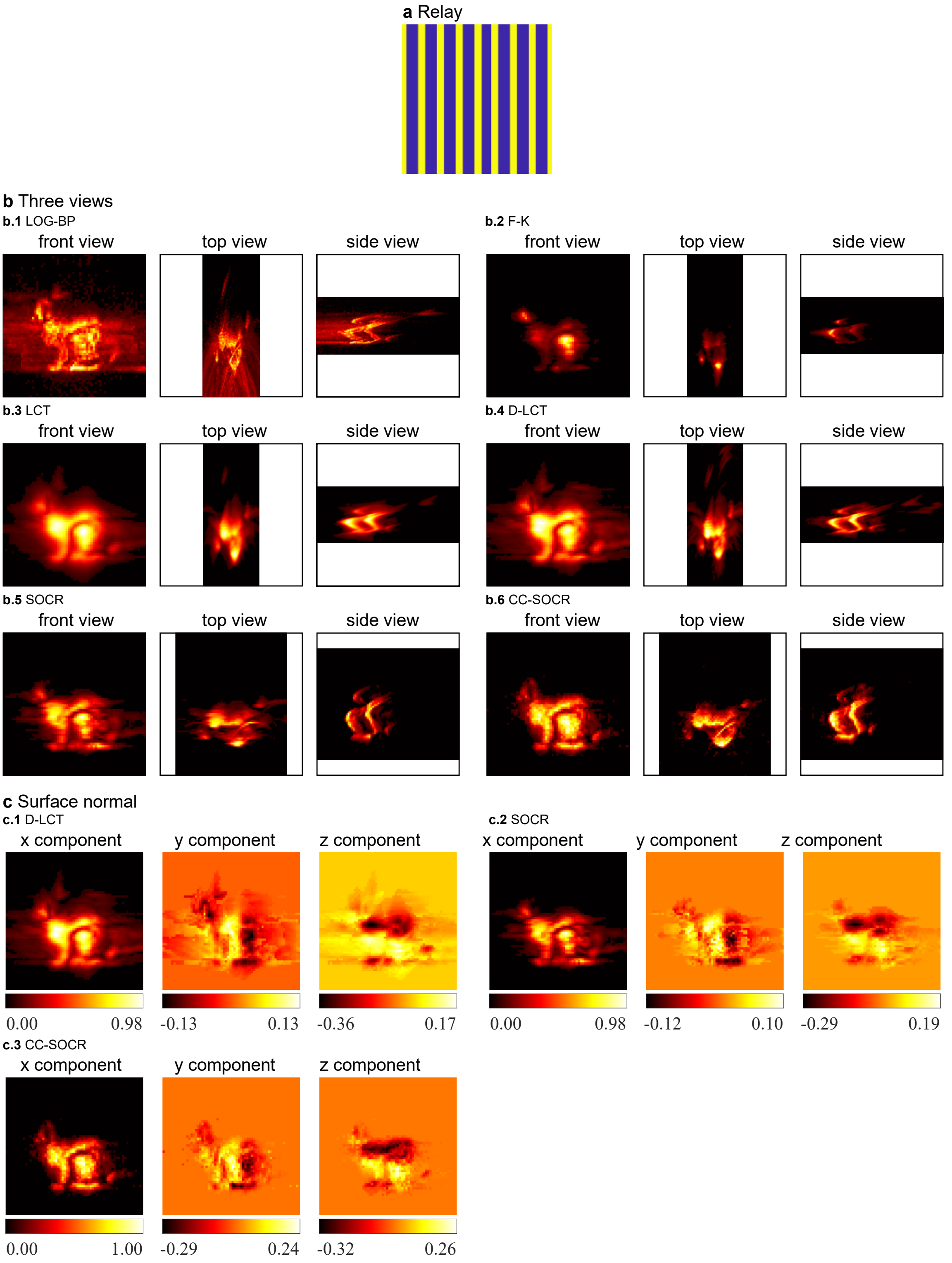

Supplementary Figure 2 Reconstructions of the bunny with vertical bars as the relay (confocal, synthetic signal). a The relay is made up of 9 equispaced vertical bars. **b** Three views of the reconstructions. For all methods, the same reconstruction domain is shown. For the LOG-BP, F-K, LCT, and D-LCT methods, the length of the voxels in the depth direction is 0.125 cm. For the SOCR and CC-SOCR methods, the length of voxels in the depth direction is 0.25 cm. **c** The reconstructed surface normal of the D-LCT, SOCR and CC-SOCR methods is shown in the form of three components. The x, y and z components show values of the directional albedo in the depth, horizontal and vertical directions, respectively.

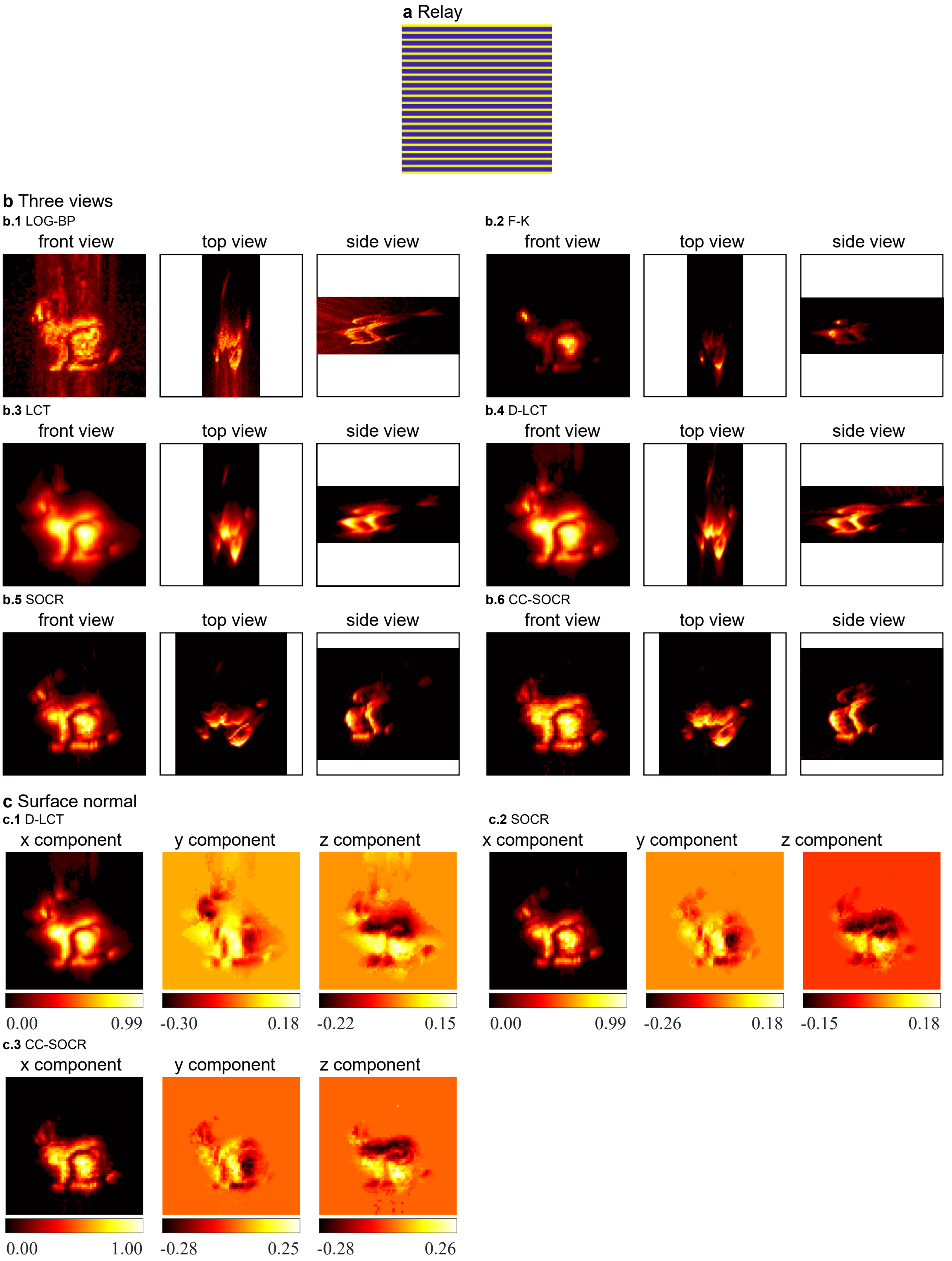

Supplementary Figure 3 Reconstructions of the bunny with horizontal bars as the relay (confocal, synthetic signal). a The relay is made up of 22 equispaced horizontal bars. **b** Three views of the reconstructions. For all methods, the same reconstruction domain is shown. For the LOG-BP, F-K, LCT, and D-LCT methods, the length of the voxels in the depth direction is 0.125 cm. For the SOCR and CC-SOCR methods, the length of voxels in the depth direction is 0.25 cm. **c** The reconstructed surface normal of the D-LCT, SOCR and CC-SOCR methods is shown in the form of three components. The x, y and z components show values of the directional albedo in the depth, horizontal and vertical directions, respectively.

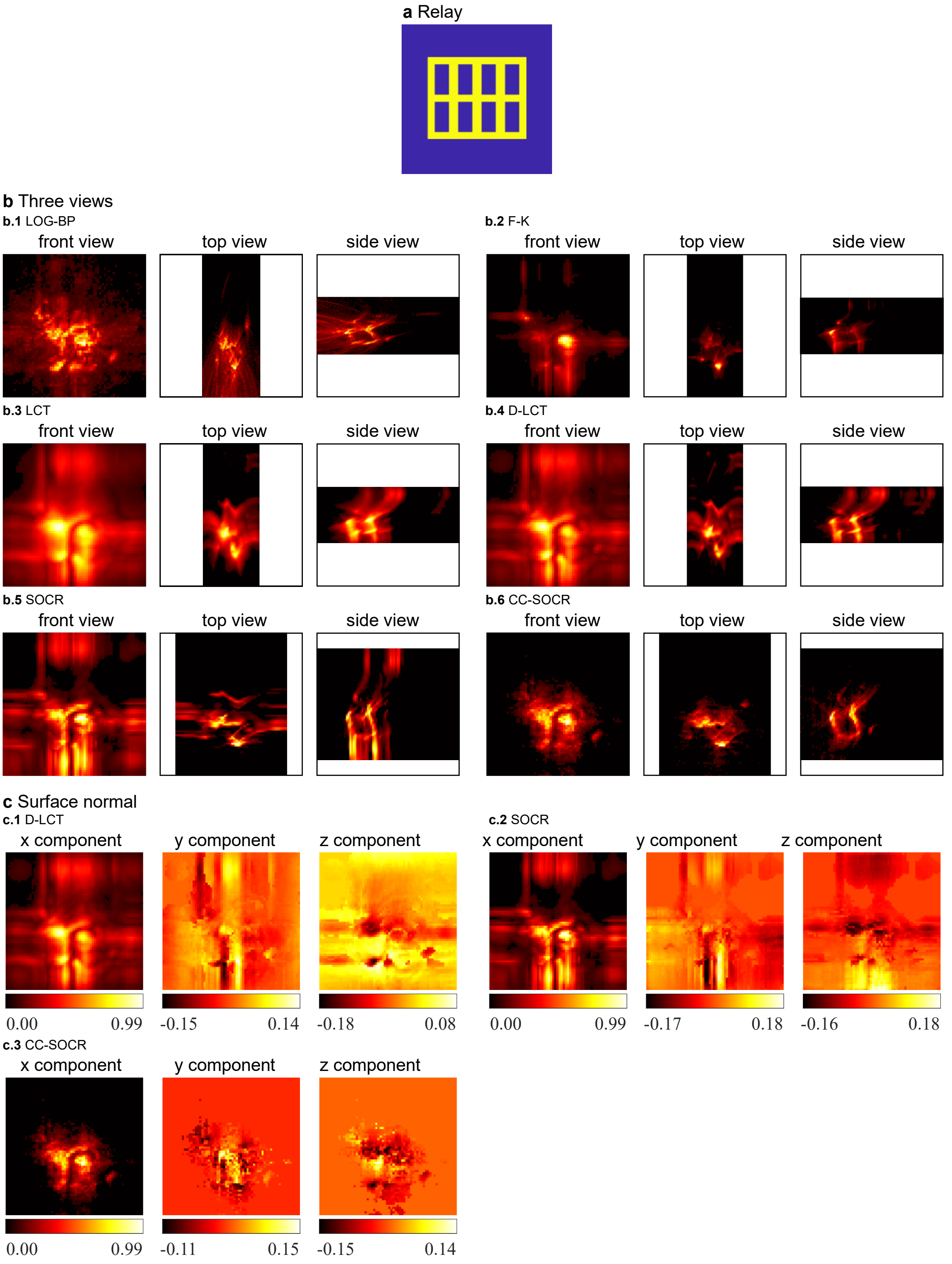

Supplementary Figure 4 Reconstructions of the bunny with an array of window edges as the relay (confocal, synthetic signal). a The relay is an array of window edges. **b** Three views of the reconstructions. For all methods, the same reconstruction domain is shown. For the LOG-BP, F-K, LCT, and D-LCT methods, the length of the voxels in the depth direction is 0.125 cm. For the SOCR and CC-SOCR methods, the length of voxels in the depth direction is 0.25 cm. **c** The reconstructed surface normal of the D-LCT, SOCR and CC-SOCR methods is shown in the form of three components. The x, y and z components show values of the directional albedo in the depth, horizontal and vertical directions, respectively.

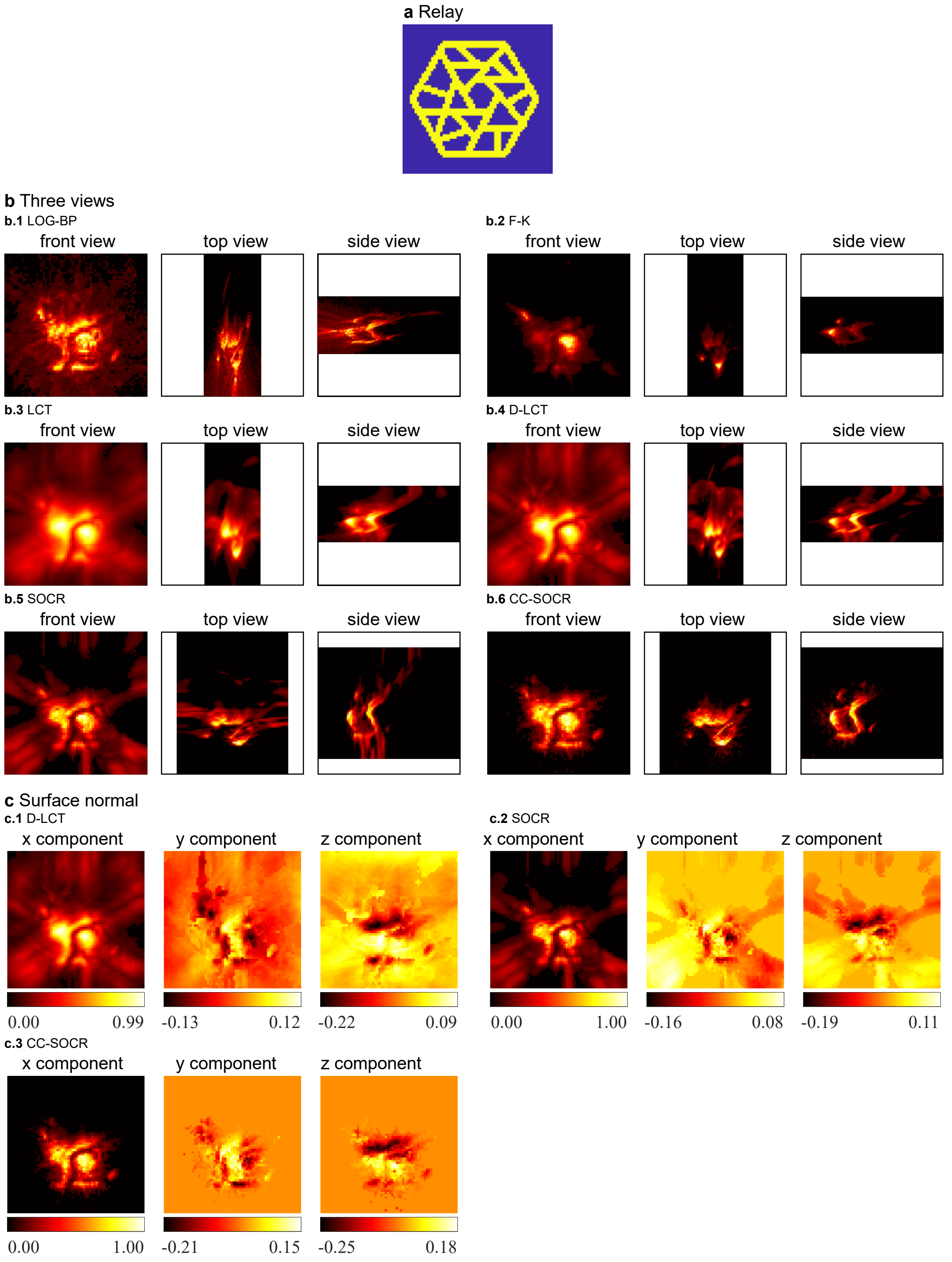

Supplementary Figure 5 Reconstructions of the bunny with an irregular relay surface (confocal, synthetic signal). a The relay is a set of several sticks sparsely and randomly distributed. **b** Three views of the reconstructions. For all methods, the same reconstruction domain is shown. For the LOG-BP, F-K, LCT, and D-LCT methods, the length of the voxels in the depth direction is 0.125 cm. For the SOCR and CC-SOCR methods, the length of voxels in the depth direction is 0.25 cm. **c** The reconstructed surface normal of the D-LCT, SOCR and CC-SOCR methods is shown in the form of three components. The x, y and z components show values of the directional albedo in the depth, horizontal and vertical directions, respectively.

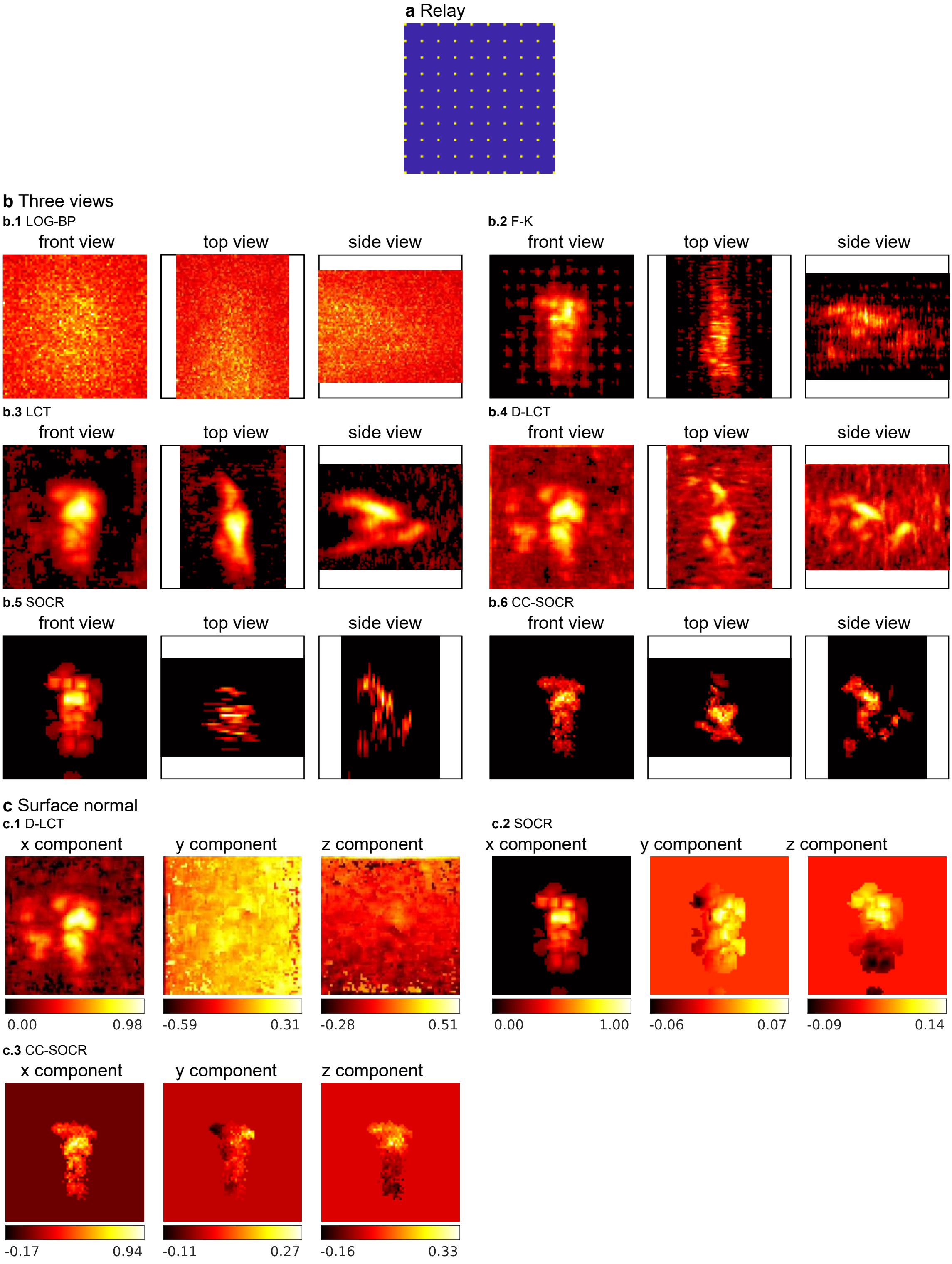

Supplementary Figure 6 Reconstructions of the statue with 10 × 10 measurements (confocal, measured signal). a Confocal signals are measured at 10 × 10 focal points. **b** Three views of the reconstructions. For all methods, the same reconstruction domain is shown. For the LOG-BP, F-K, LCT, and D-LCT methods, the length of the voxels in the depth direction is 0.48 cm. For the SOCR and CC-SOCR methods, the length of voxels in the depth direction is 0.96 cm. **c** The reconstructed surface normal of the D-LCT, SOCR and CC-SOCR methods is shown in the form of three components. The x, y and z components show values of the directional albedo in the depth, horizontal and vertical directions, respectively.

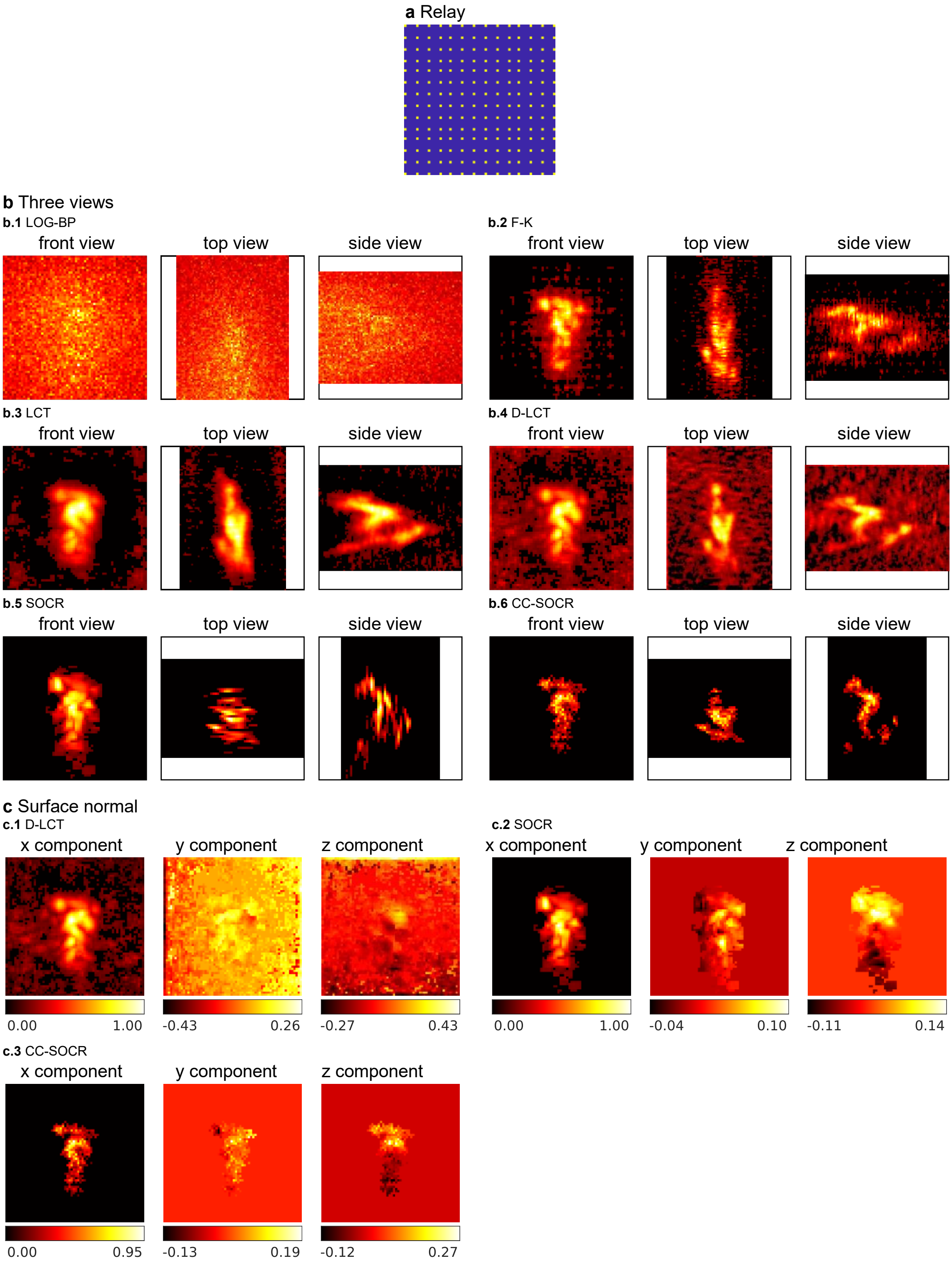

Supplementary Figure 7 Reconstructions of the statue with 14 × 14 measurements (confocal, measured signal). a Confocal signals are measured at 14 × 14 focal points. **b** Three views of the reconstructions. For all methods, the same reconstruction domain is shown. For the LOG-BP, F-K, LCT, and D-LCT methods, the length of the voxels in the depth direction is 0.48 cm. For the SOCR and CC-SOCR methods, the length of voxels in the depth direction is 0.96 cm. **c** The reconstructed surface normal of the D-LCT, SOCR and CC-SOCR methods is shown in the form of three components. The x, y and z components show values of the directional albedo in the depth, horizontal and vertical directions, respectively.

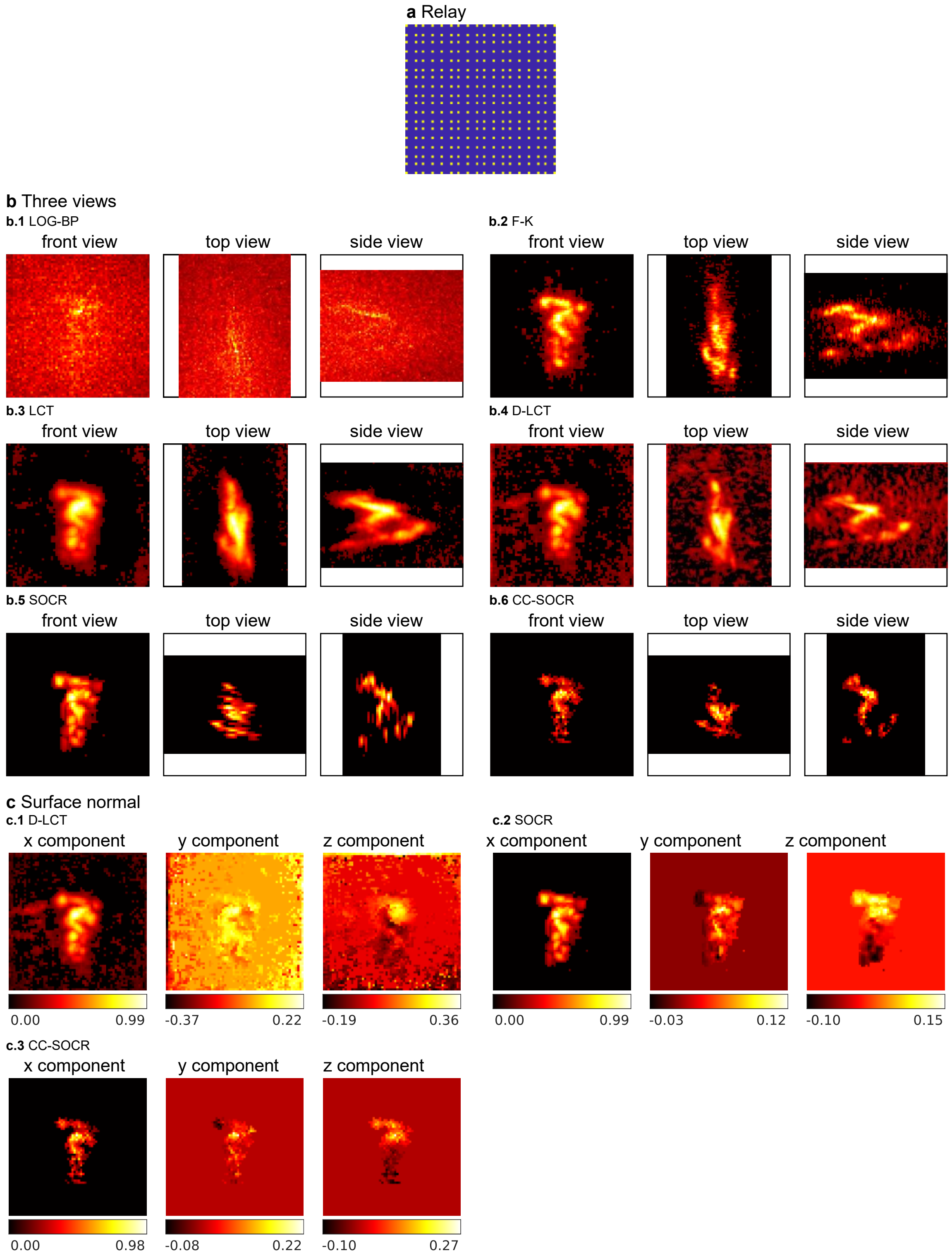

Supplementary Figure 8 Reconstructions of the statue with 18 × 18 measurements (confocal, measured signal). a Confocal signals are measured at 18 × 18 focal points. **b** Three views of the reconstructions. For all methods, the same reconstruction domain is shown. For the LOG-BP, F-K, LCT, and D-LCT methods, the length of the voxels in the depth direction is 0.48 cm. For the SOCR and CC-SOCR methods, the length of voxels in the depth direction is 0.96 cm. **c** The reconstructed surface normal of the D-LCT, SOCR and CC-SOCR methods is shown in the form of three components. The x, y and z components show values of the directional albedo in the depth, horizontal and vertical directions, respectively.

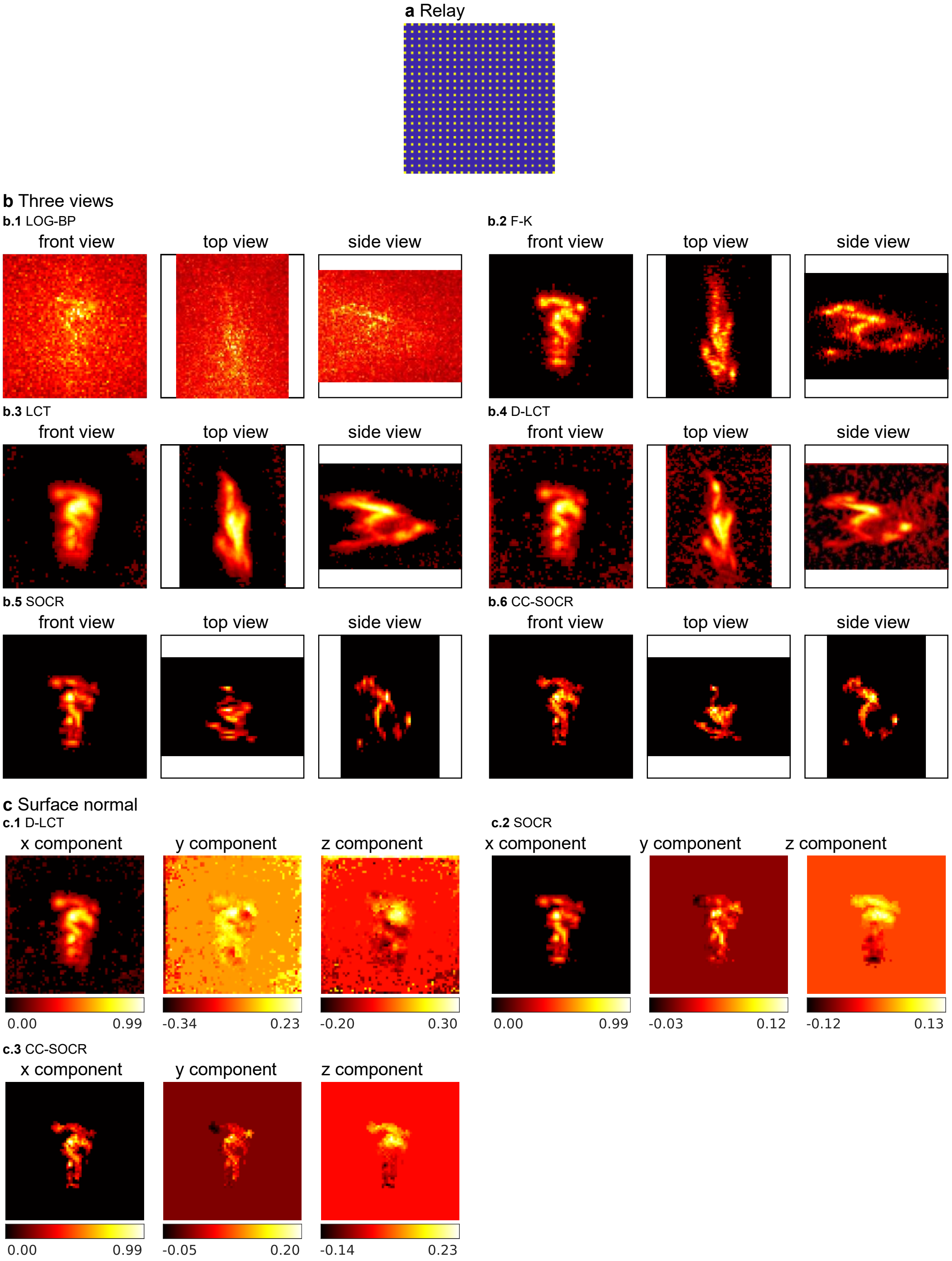

Supplementary Figure 9 Reconstructions of the statue with 22 × 22 measurements (confocal, measured signal). a Confocal signals are measured at 22 × 22 focal points. **b** Three views of the reconstructions. For all methods, the same reconstruction domain is shown. For the LOG-BP, F-K, LCT, and D-LCT methods, the length of the voxels in the depth direction is 0.48 cm. For the SOCR and CC-SOCR methods, the length of voxels in the depth direction is 0.96 cm. **c** The reconstructed surface normal of the D-LCT, SOCR and CC-SOCR methods is shown in the form of three components. The x, y and z components show values of the directional albedo in the depth, horizontal and vertical directions, respectively.

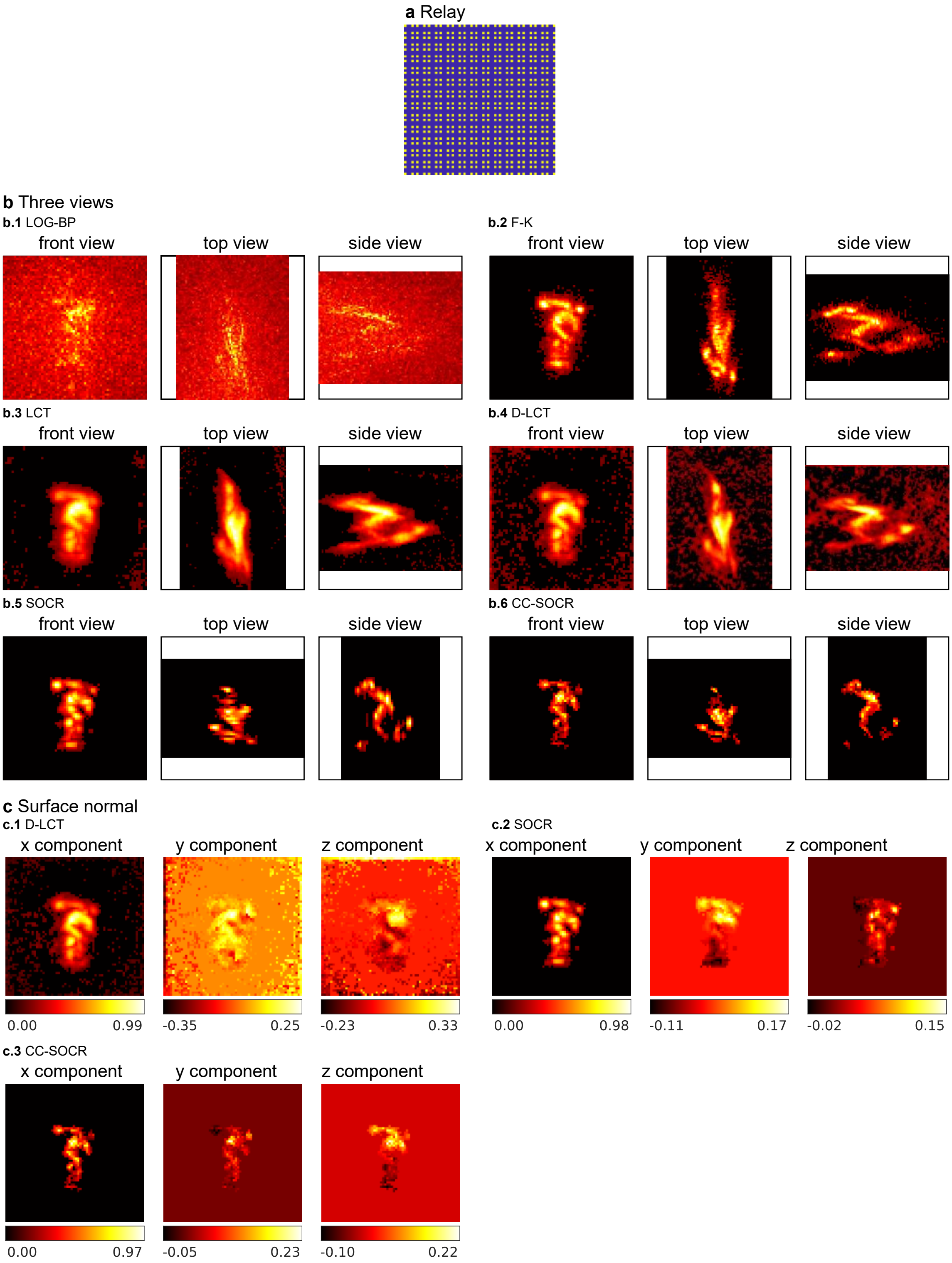

Supplementary Figure 10 Reconstructions of the statue with 26 × 26 measurements (confocal, measured signal). a Confocal signals are measured at 26 × 26 focal points. **b** Three views of the reconstructions. For all methods, the same reconstruction domain is shown. For the LOG-BP, F-K, LCT, and D-LCT methods, the length of the voxels in the depth direction is 0.48 cm. For the SOCR and CC-SOCR methods, the length of voxels in the depth direction is 0.96 cm. **c** The reconstructed surface normal of the D-LCT, SOCR and CC-SOCR methods is shown in the form of three components. The x, y and z components show values of the directional albedo in the depth, horizontal and vertical directions, respectively.

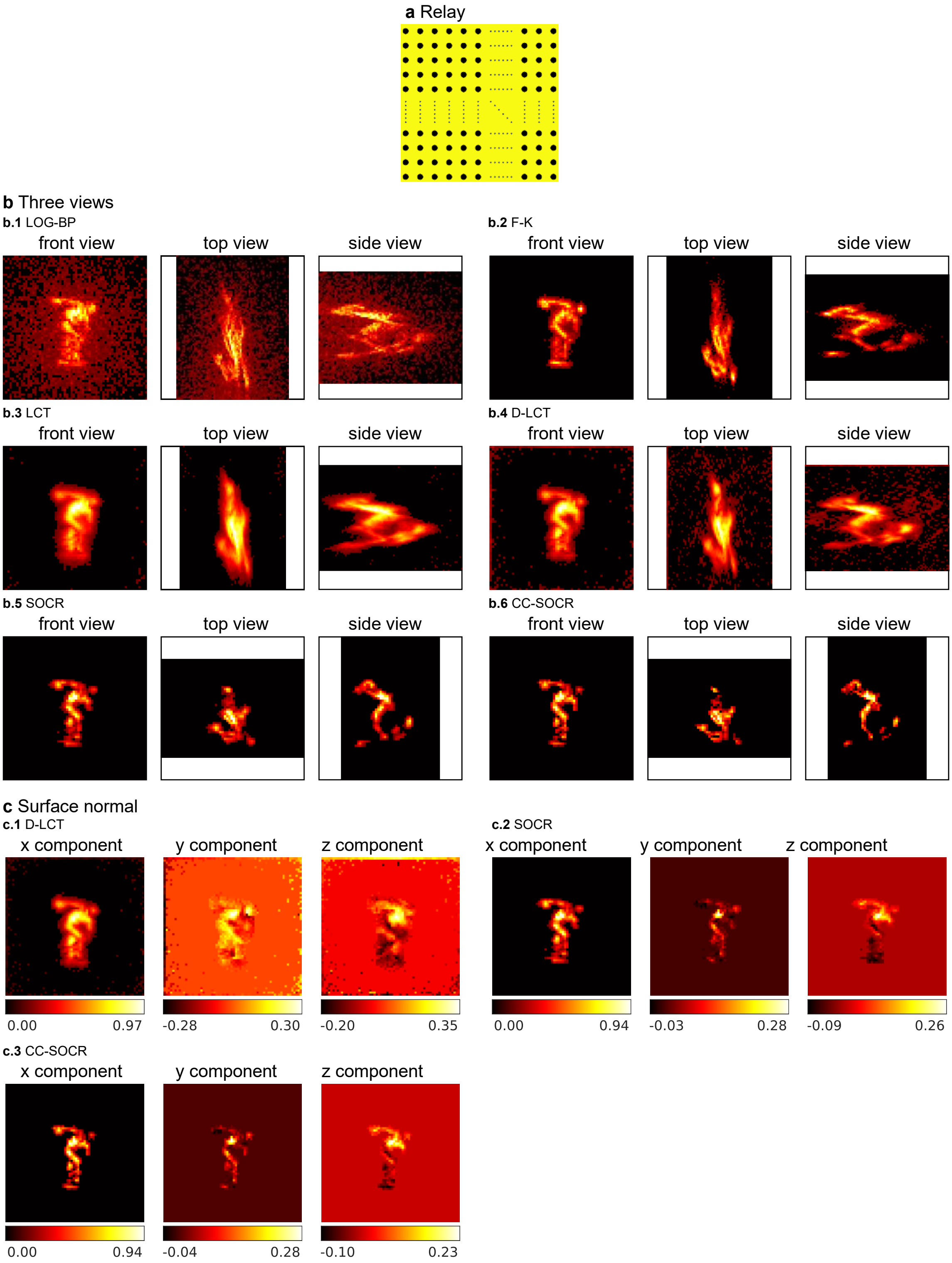

Supplementary Figure 11 Reconstructions of the statue with 64 × 64 measurements (confocal, measured signal). a Confocal signals are measured at 64 × 64 focal points. **b** Three views of the reconstructions. For all methods, the same reconstruction domain is shown. For the LOG-BP, F-K, LCT, and D-LCT methods, the length of the voxels in the depth direction is 0.48 cm. For the SOCR and CC-SOCR methods, the length of voxels in the depth direction is 0.96 cm. **c** The reconstructed surface normal of the D-LCT, SOCR and CC-SOCR methods is shown in the form of three components. The x, y and z components show values of the directional albedo in the depth, horizontal and vertical directions, respectively.

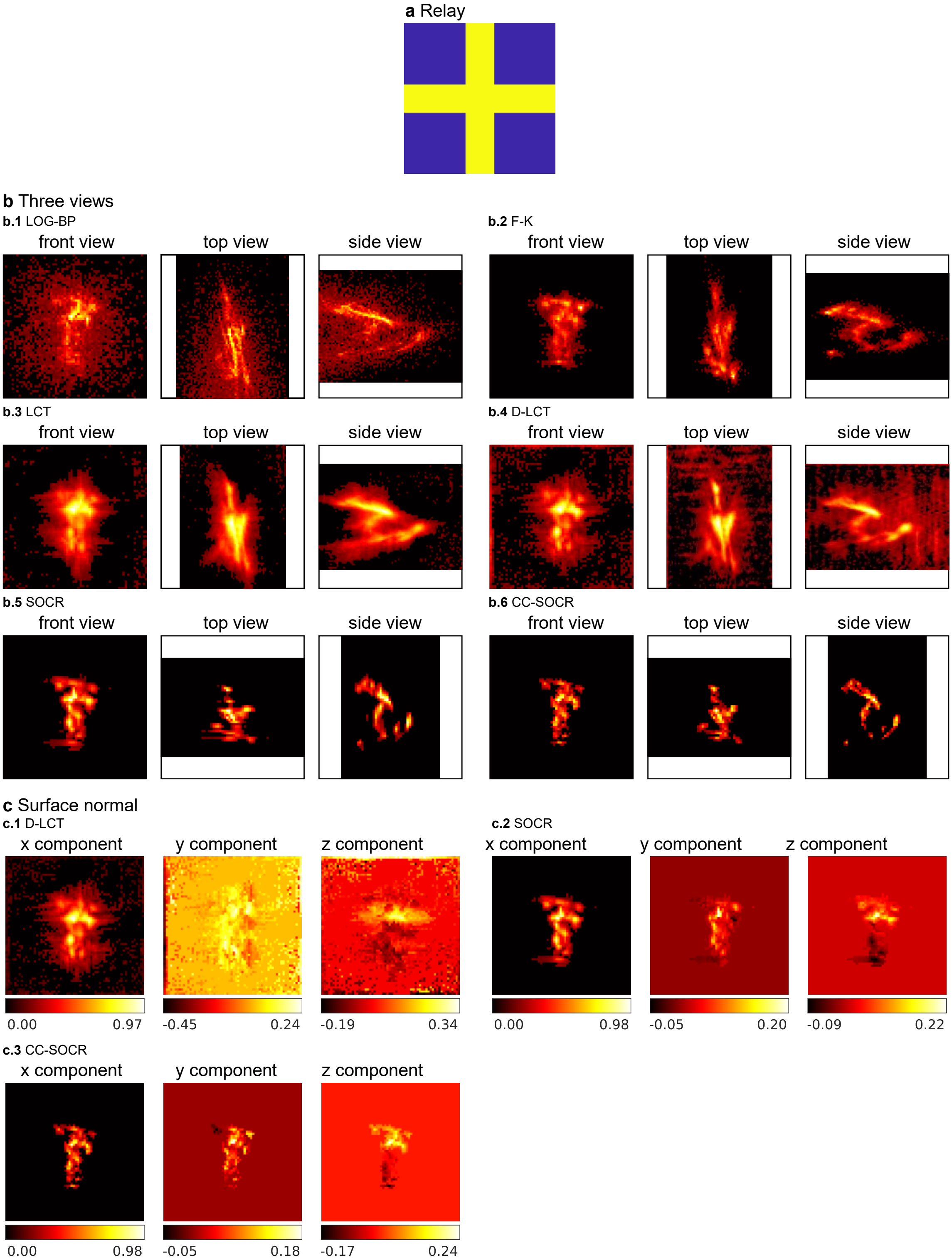

Supplementary Figure 12 Reconstructions of the statue with a cross-shaped relay surface (confocal, measured signal). a Confocal signals are measured in the yellow region, which consists of 1392 focal points. **b** Three views of the reconstructions. For all methods, the same reconstruction domain is shown. For the LOG-BP, F-K, LCT, and D-LCT methods, the length of the voxels in the depth direction is 0.48 cm. For the SOCR and CC-SOCR methods, the length of voxels in the depth direction is 0.96 cm. **c** The reconstructed surface normal of the D-LCT, SOCR and CC-SOCR methods is shown in the form of three components. The x, y and z components show values of the directional albedo in the depth, horizontal and vertical directions, respectively.

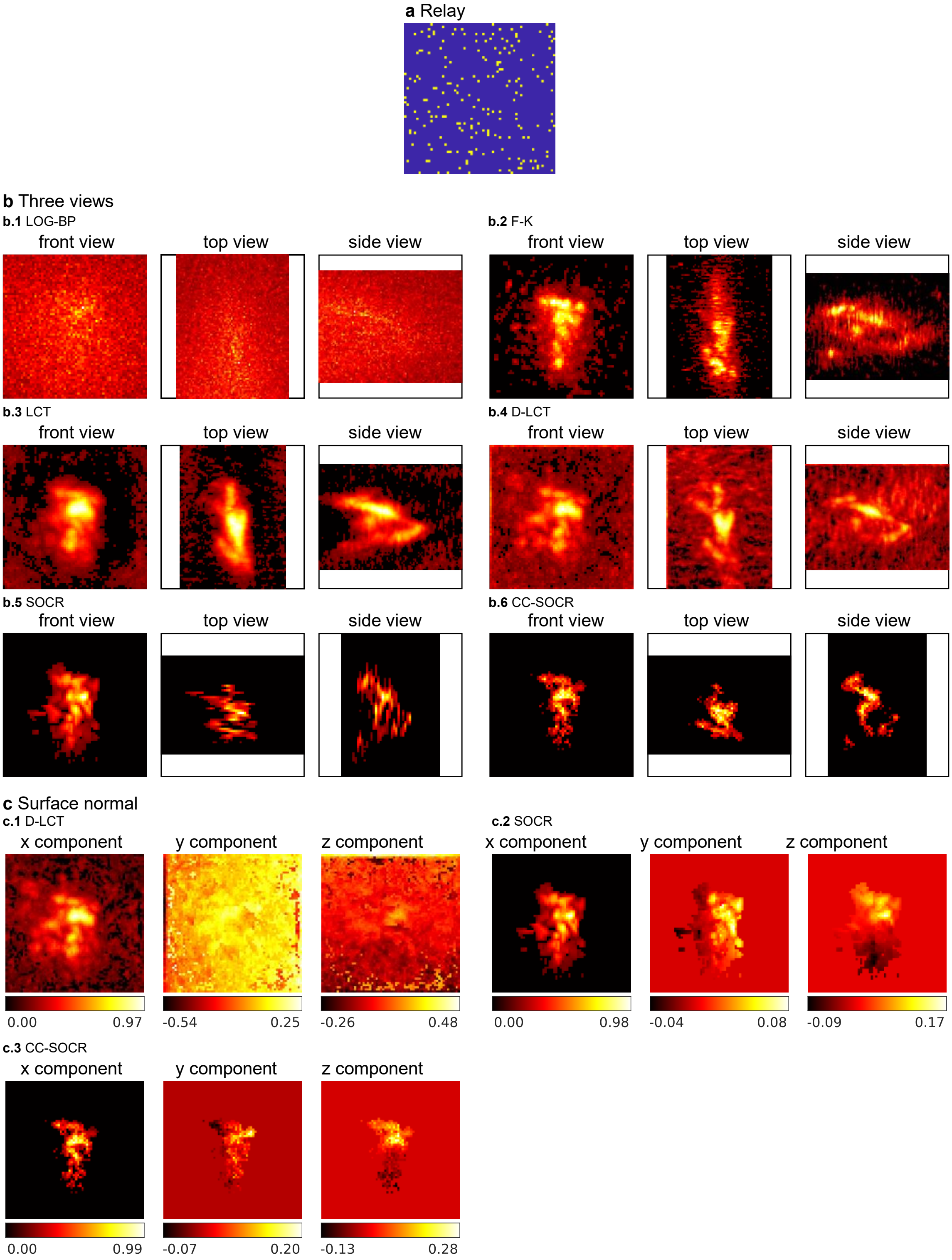

Supplementary Figure 13 Reconstructions of the statue with signals measured at 200 randomly chosen focal points (confocal, measured signal). A The confocal signal is measured at 200 randomly chosen focal points. **b** Three views of the reconstructions. For all methods, the same reconstruction domain is shown. For the LOG-BP, F-K, LCT, and D-LCT methods, the length of the voxels in the depth direction is 0.48 cm. For the SOCR and CC-SOCR methods, the length of voxels in the depth direction is 0.96 cm. **c** The reconstructed surface normal of the D-LCT, SOCR and CC-SOCR methods is shown in the form of three components. The x, y and z components show values of the directional albedo in the depth, horizontal and vertical directions, respectively.

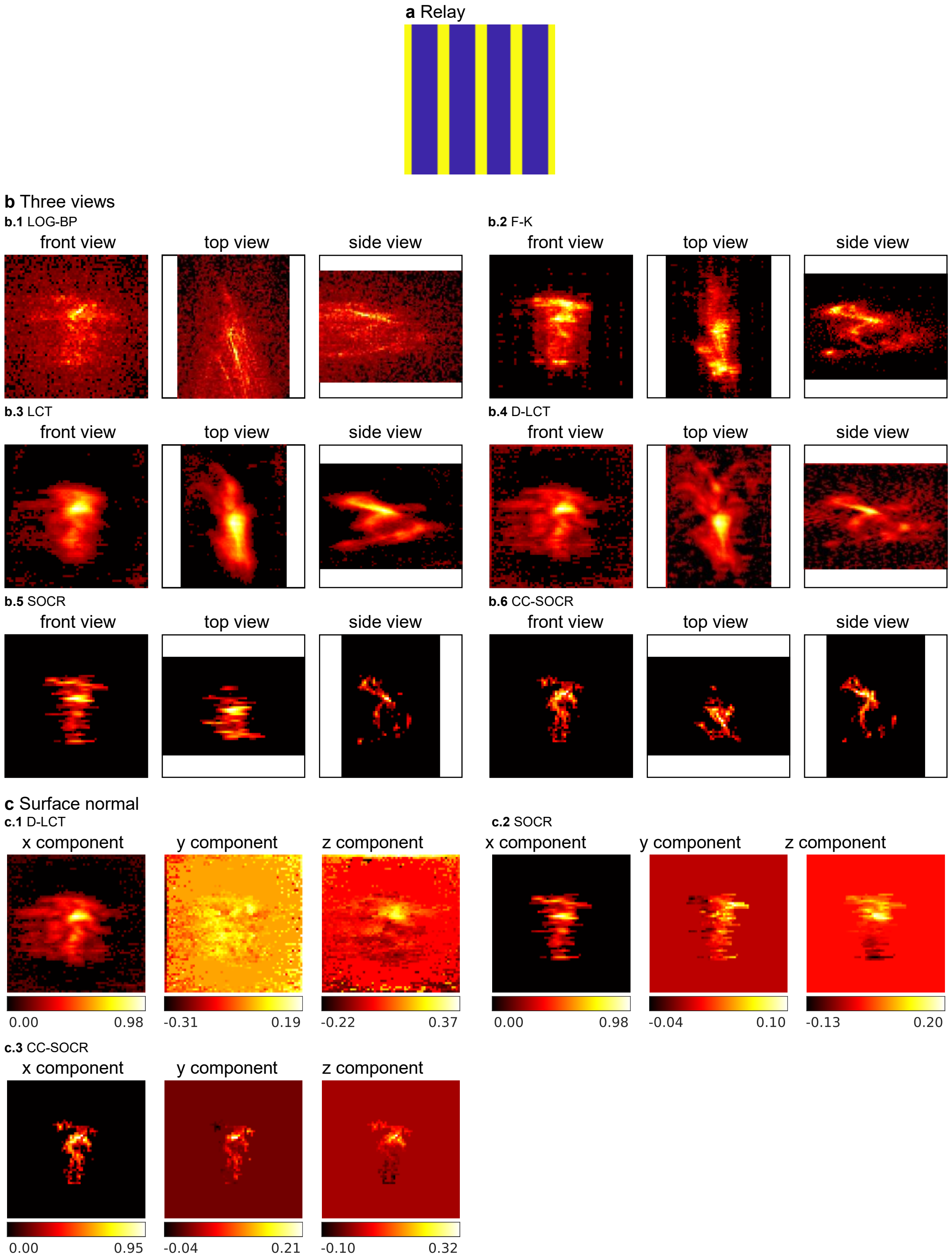

Supplementary Figure 14 Reconstructions of the statue with signals measured at vertical bars (confocal, measured signal). a The confocal signal is measured at 5 equispaced vertical bars, which contains 1344 focal points. **b** Three views of the reconstructions. For all methods, the same reconstruction domain is shown. For the LOG-BP, F-K, LCT, and D-LCT methods, the length of the voxels in the depth direction is 0.48 cm. For the SOCR and CC-SOCR methods, the length of voxels in the depth direction is 0.96 cm. **c** The reconstructed surface normal of the D-LCT, SOCR and CC-SOCR methods is shown in the form of three components. The x, y and z components show values of the directional albedo in the depth, horizontal and vertical directions, respectively.

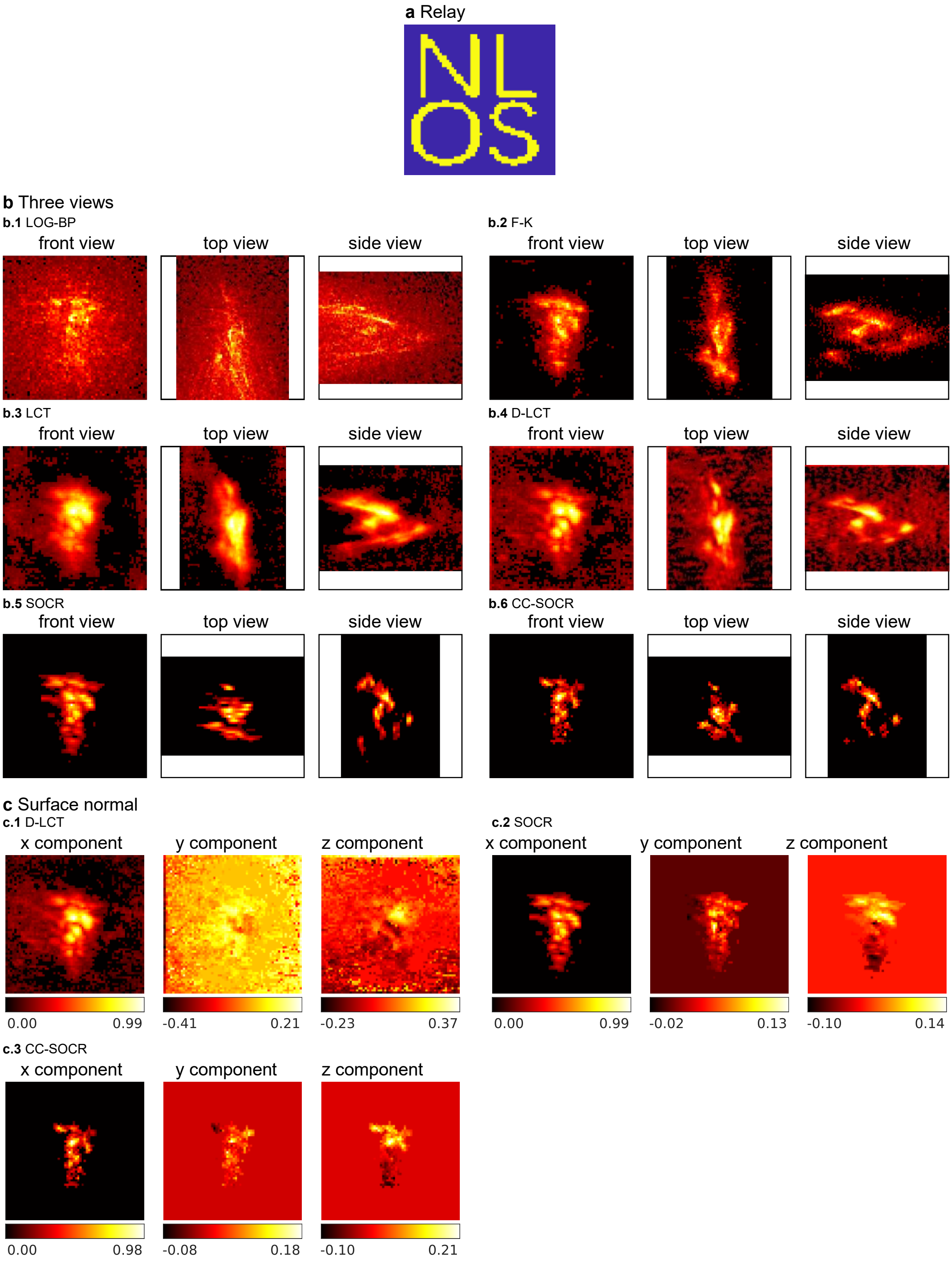

Supplementary Figure 15 Reconstructions of the statue with confocal signals measured at the letters N, L, O and S. a The relay is the region consisting of four letters N, L, O and S, which includes 825 focal points. **b** Three views of the reconstructions. For all methods, the same reconstruction domain is shown. For the LOG-BP, F-K, LCT, and D-LCT methods, the length of the voxels in the depth direction is 0.48 cm. For the SOCR and CC-SOCR methods, the length of voxels in the depth direction is 0.96 cm. **c** The reconstructed surface normal of the D-LCT, SOCR and CC-SOCR methods is shown in the form of three components. The x, y and z components show values of the directional albedo in the depth, horizontal and vertical directions, respectively.

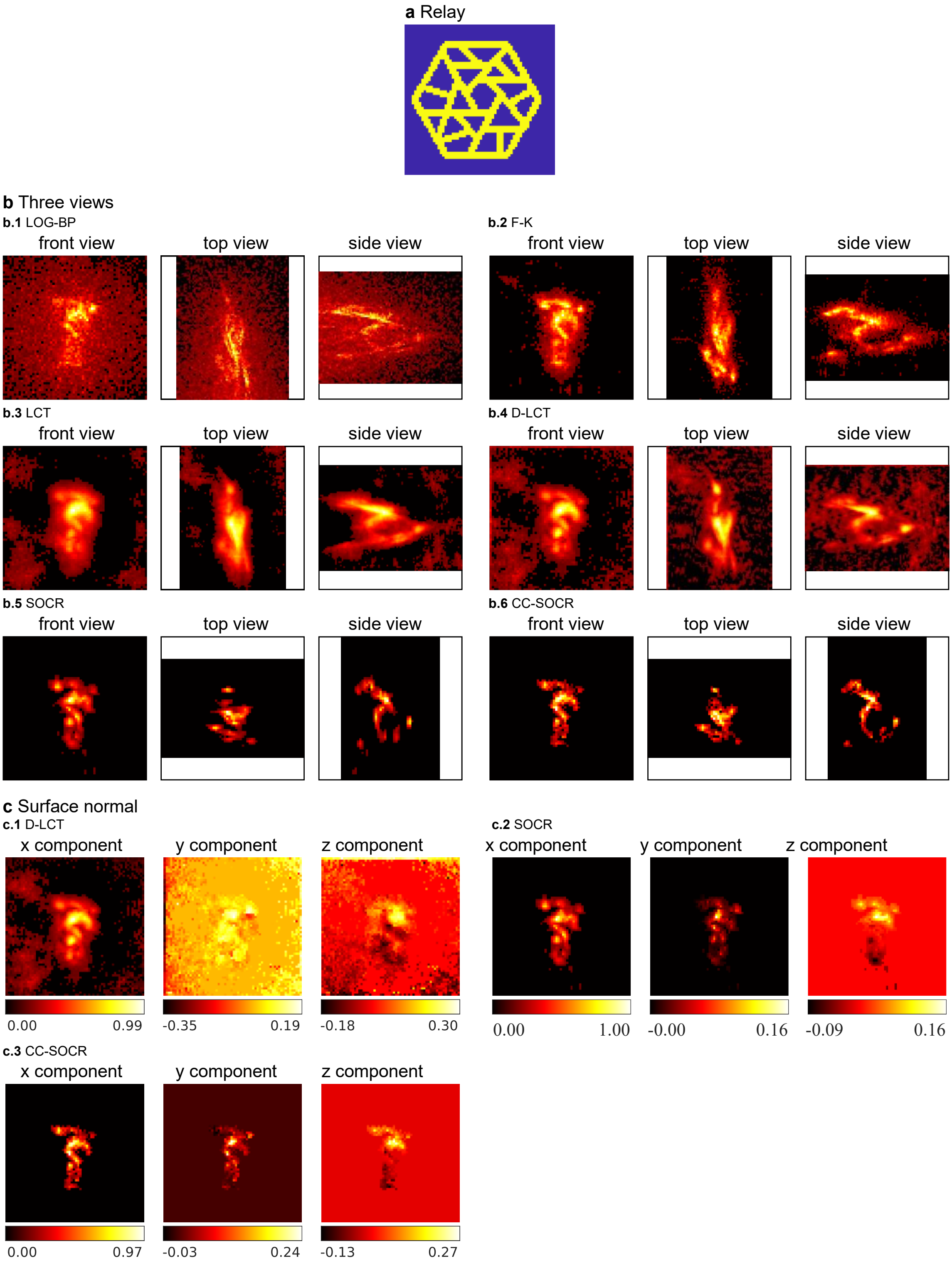

Supplementary Figure 16 Reconstructions of the statue with an irregular relay. a The relay is a set of several sticks sparsely and randomly distributed, which contains 1229 focal points. **b** Three views of the reconstructions. For all methods, the same reconstruction domain is shown. For the LOG-BP, F-K, LCT, and D-LCT methods, the length of the voxels in the depth direction is 0.48 cm. For the SOCR and CC-SOCR methods, the length of voxels in the depth direction is 0.96 cm. **c** The reconstructed surface normal of the D-LCT, SOCR and CC-SOCR methods is shown in the form of three components. The x, y and z components show values of the directional albedo in the depth, horizontal and vertical directions, respectively.

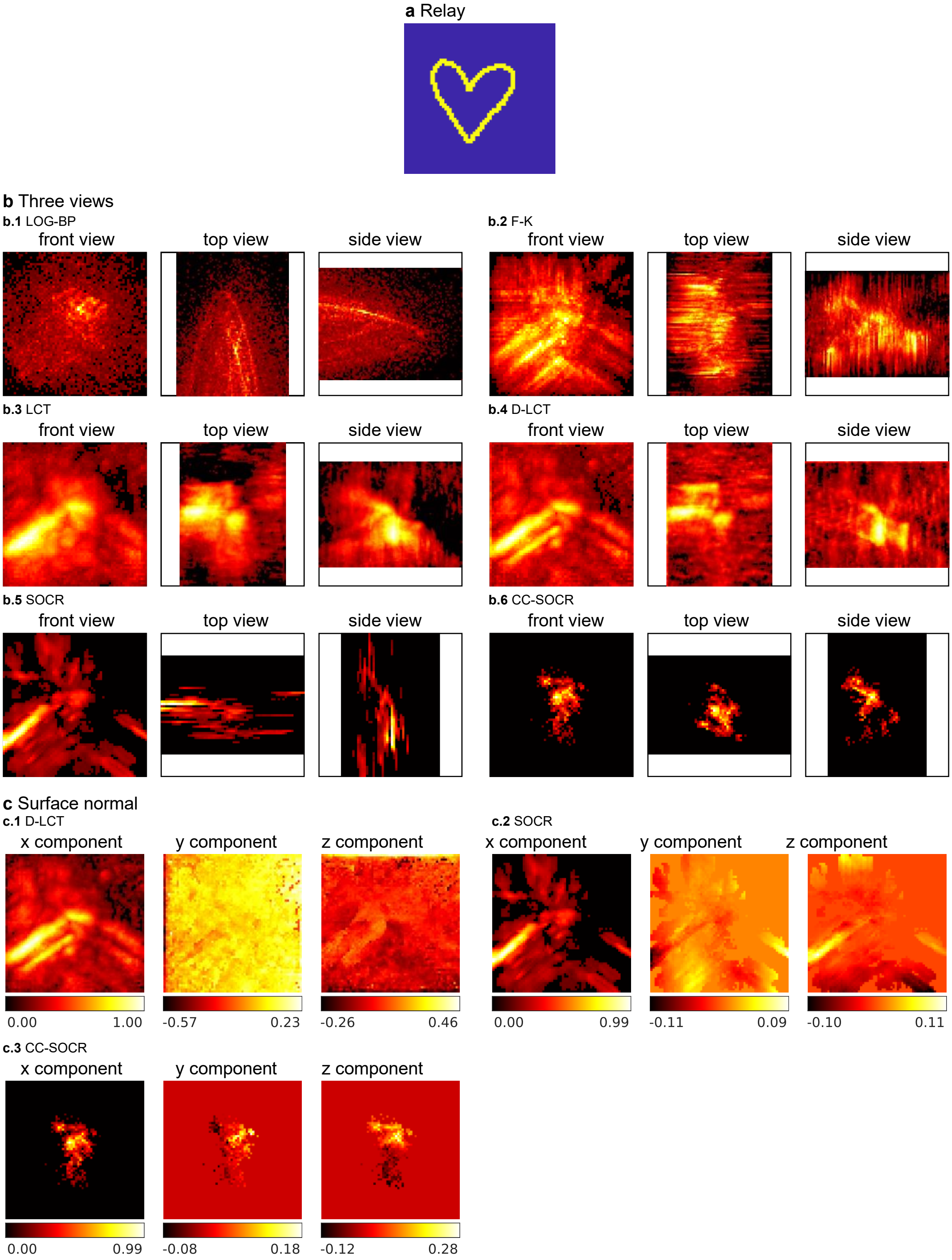

Supplementary Figure 17 Reconstructions of the statue with a heart-shaped relay surface. a The relay is a heart-shaped region, which contains 258 focal points. **b** Three views of the reconstructions. For all methods, the same reconstruction domain is shown. For the LOG-BP, F-K, LCT, and D-LCT methods, the length of the voxels in the depth direction is 0.48 cm. For the SOCR and CC-SOCR methods, the length of voxels in the depth direction is 0.96 cm. **c** The reconstructed surface normal of the D-LCT, SOCR and CC-SOCR methods is shown in the form of three components. The x, y and z components show values of the directional albedo in the depth, horizontal and vertical directions, respectively.

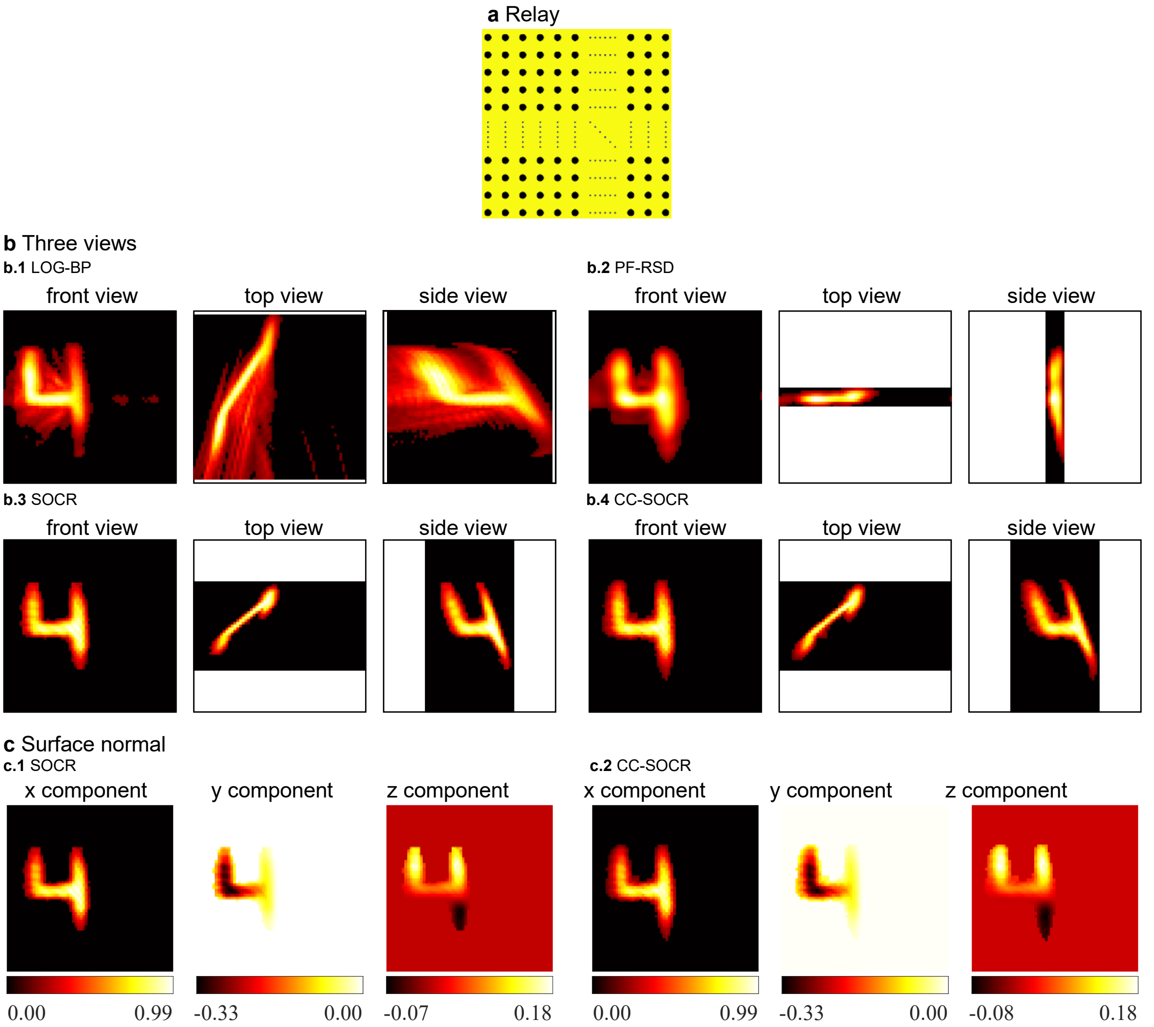

Supplementary Figure 18 Reconstructions of the figure 4 with 64 × 64 measurements (non-confocal, measured signal). a 64 × 64 focal points are raster scanned. **b** Three views of the reconstructions. For all methods, the same reconstruction domain is shown. For the LOG-BP, PF-RSD, SOCR and CC-SOCR methods, the lengths of the voxels in the depth direction are 0.24 cm, 1.87 cm, 0.48 cm and 0.48 cm. **c** The reconstructed surface normal of the SOCR and CC-SOCR methods is shown in the form of three components. The x, y and z components show values of the directional albedo in the depth, horizontal and vertical directions, respectively.

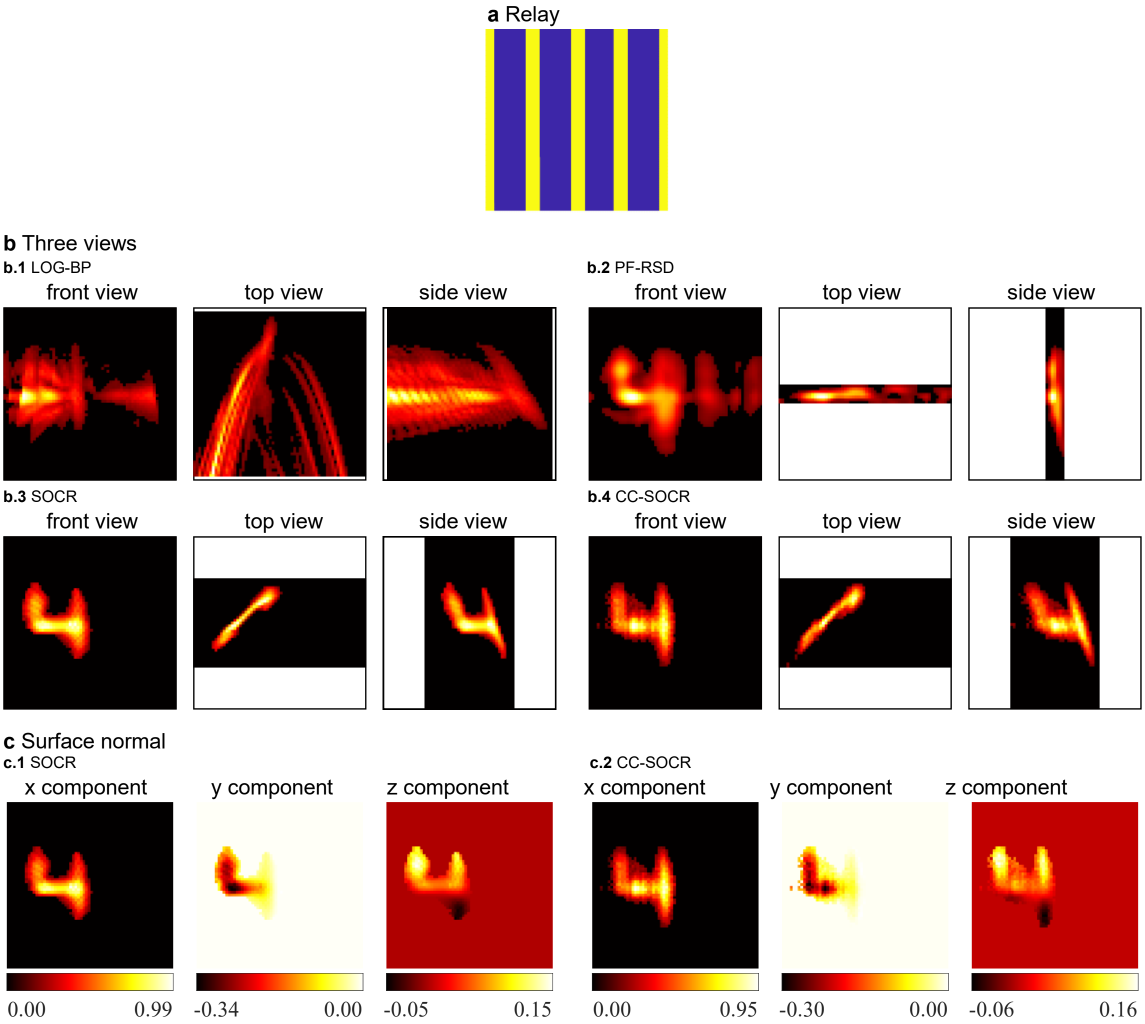

Supplementary Figure 19 Reconstructions of the figure 4 with a vertical illumination pattern (non-confocal, measured signal). a The illumination points are in the yellow region. **b** Three views of the reconstructions. For all methods, the same reconstruction domain is shown. For the LOG-BP, PF-RSD, SOCR and CC-SOCR methods, the lengths of the voxels in the depth direction are 0.24 cm, 1.87 cm, 0.48 cm and 0.48 cm. **c** The reconstructed surface normal of the SOCR and CC-SOCR methods is shown in the form of three components. The x, y and z components show values of the directional albedo in the depth, horizontal and vertical directions, respectively.

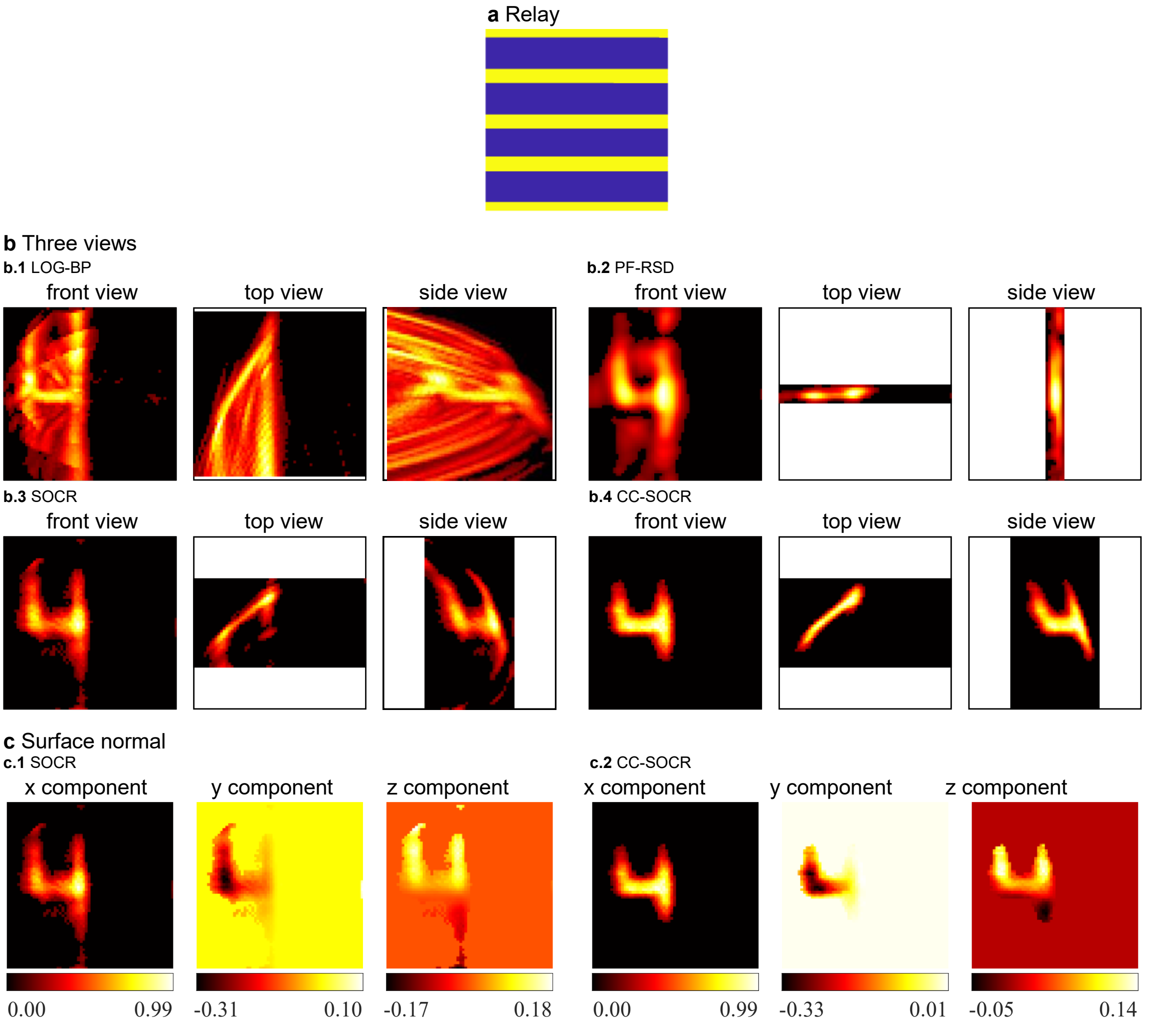

Supplementary Figure 20 Reconstructions of the figure 4 with a horizontal illumination pattern (non-confocal, measured signal). a The illumination points are in the yellow region. **b** Three views of the reconstructions. For all methods, the same reconstruction domain is shown. For the LOG-BP, PF-RSD, SOCR and CC-SOCR methods, the lengths of the voxels in the depth direction are 0.24 cm, 1.87 cm, 0.48 cm and 0.48 cm. **c** The reconstructed surface normal of the SOCR and CC-SOCR methods is shown in the form of three components. The x, y and z components show values of the directional albedo in the depth, horizontal and vertical directions, respectively.

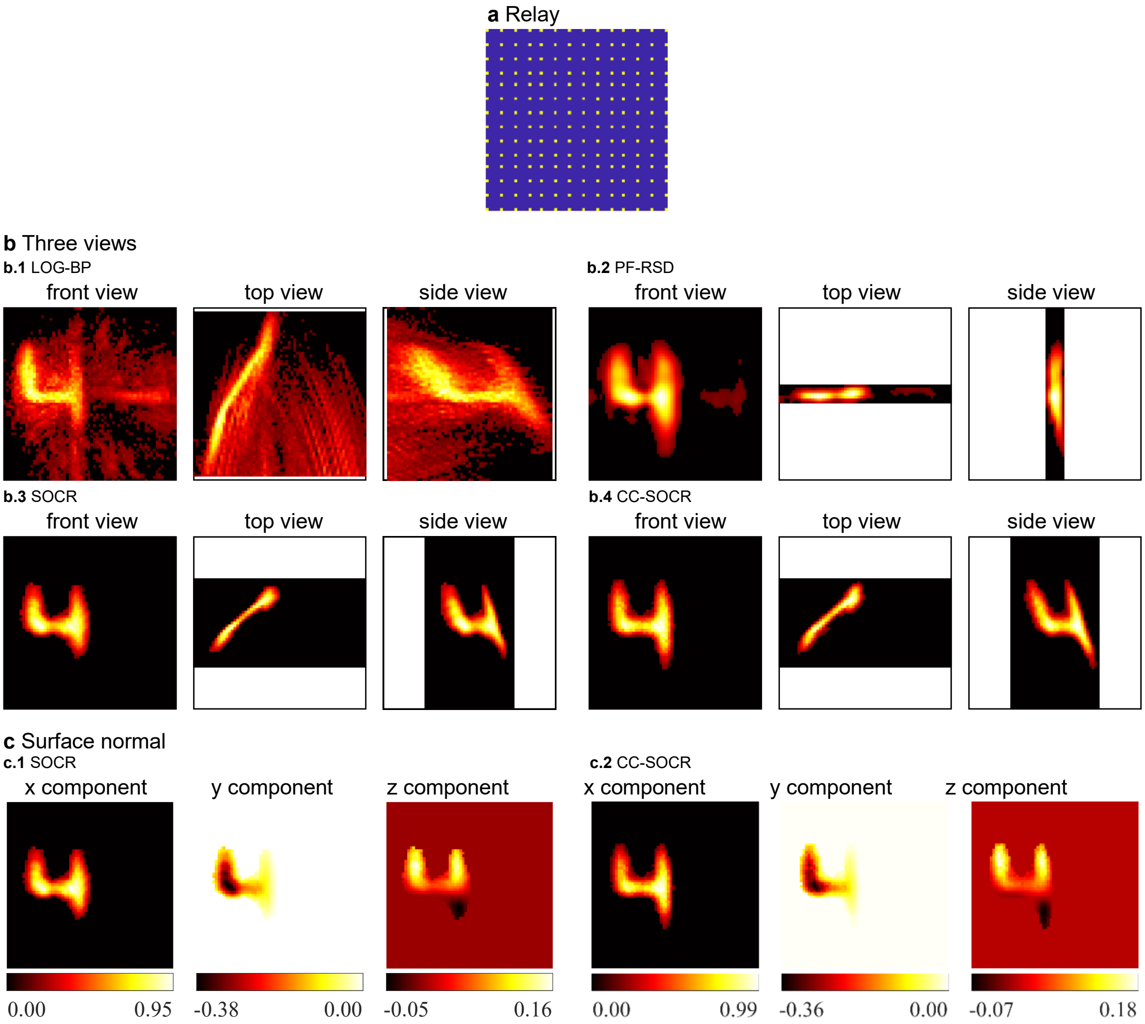

Supplementary Figure 21 Reconstructions of the figure 4 with 14 × 14 illumination points (non-confocal, measured signal). a The illumination points are shown in yellow. **b** Three views of the reconstructions. For all methods, the same reconstruction domain is shown. For the LOG-BP, PF-RSD, SOCR and CC-SOCR methods, the lengths of the voxels in the depth direction are 0.24 cm, 1.87 cm, 0.48 cm and 0.48 cm. **c** The reconstructed surface normal of the SOCR and CC-SOCR methods is shown in the form of three components. The x, y and z components show values of the directional albedo in the depth, horizontal and vertical directions, respectively.

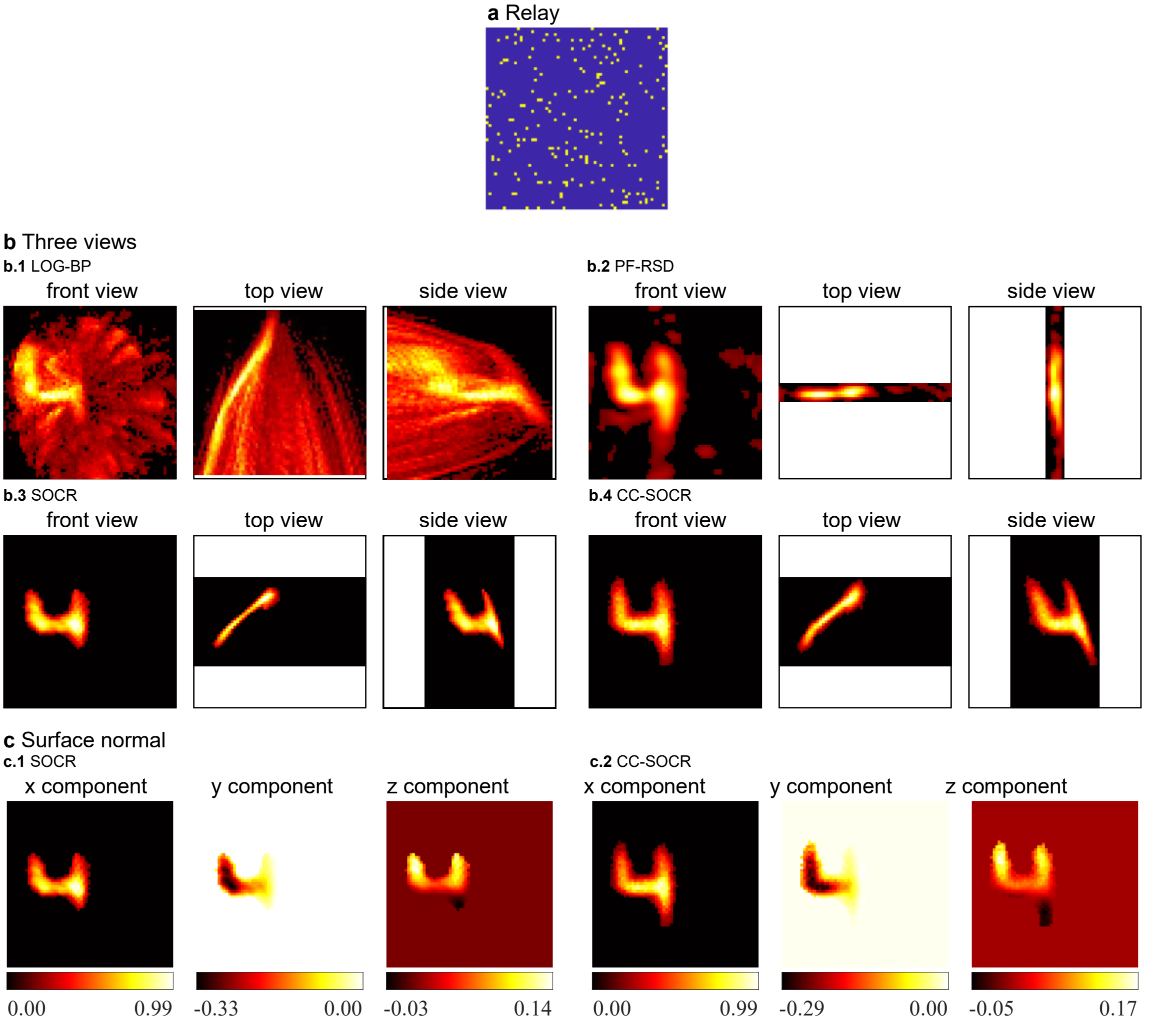

Supplementary Figure 22 Reconstructions of the figure 4 with 200 randomly distributed illumination points (non-confocal, measured signal). a The illumination points are shown in yellow. **b** Three views of the reconstructions. For all methods, the same reconstruction domain is shown. For the LOG-BP, PF-RSD, SOCR and CC-SOCR methods, the lengths of the voxels in the depth direction are 0.24 cm, 1.87 cm, 0.48 cm and 0.48 cm. **c** The reconstructed surface normal of the SOCR and CC-SOCR methods is shown in the form of three components. The x, y and z components show values of the directional albedo in the depth, horizontal and vertical directions, respectively.

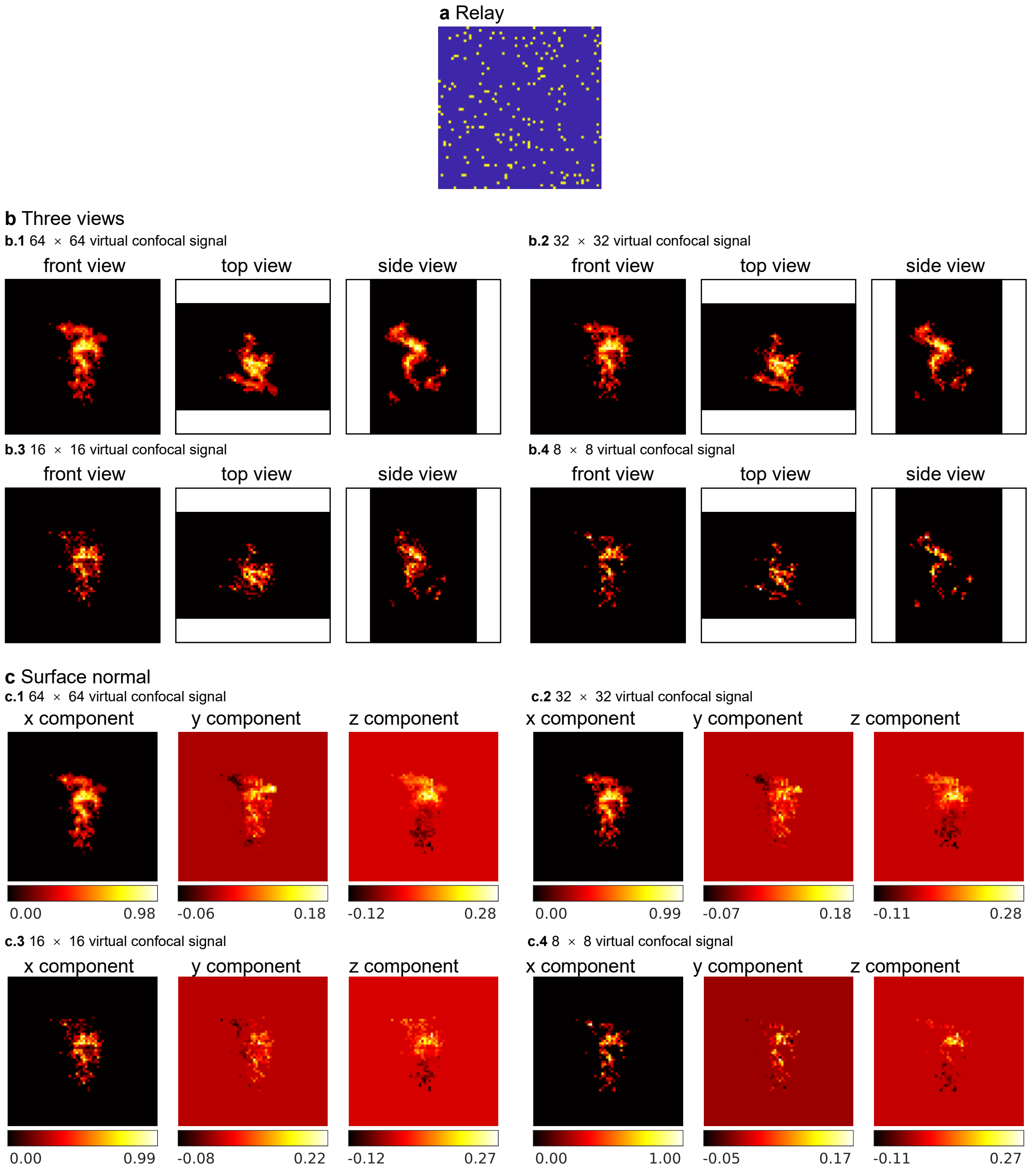

Supplementary Figure 23 Reconstructions of the statue with different sizes of the virtual confocal signals. (confocal, measured signal). a Confocal signals are measured at 200 randomly distributed focal points. **b** Three views of the reconstructions. The virtual confocal signals of different sizes are introduced. The reconstruction quality decreases with the size of the virtual confocal signal. **c** The reconstructed surface normal is shown in the form of three components. The x, y and z components show values of the directional albedo in the depth, horizontal and vertical directions, respectively.

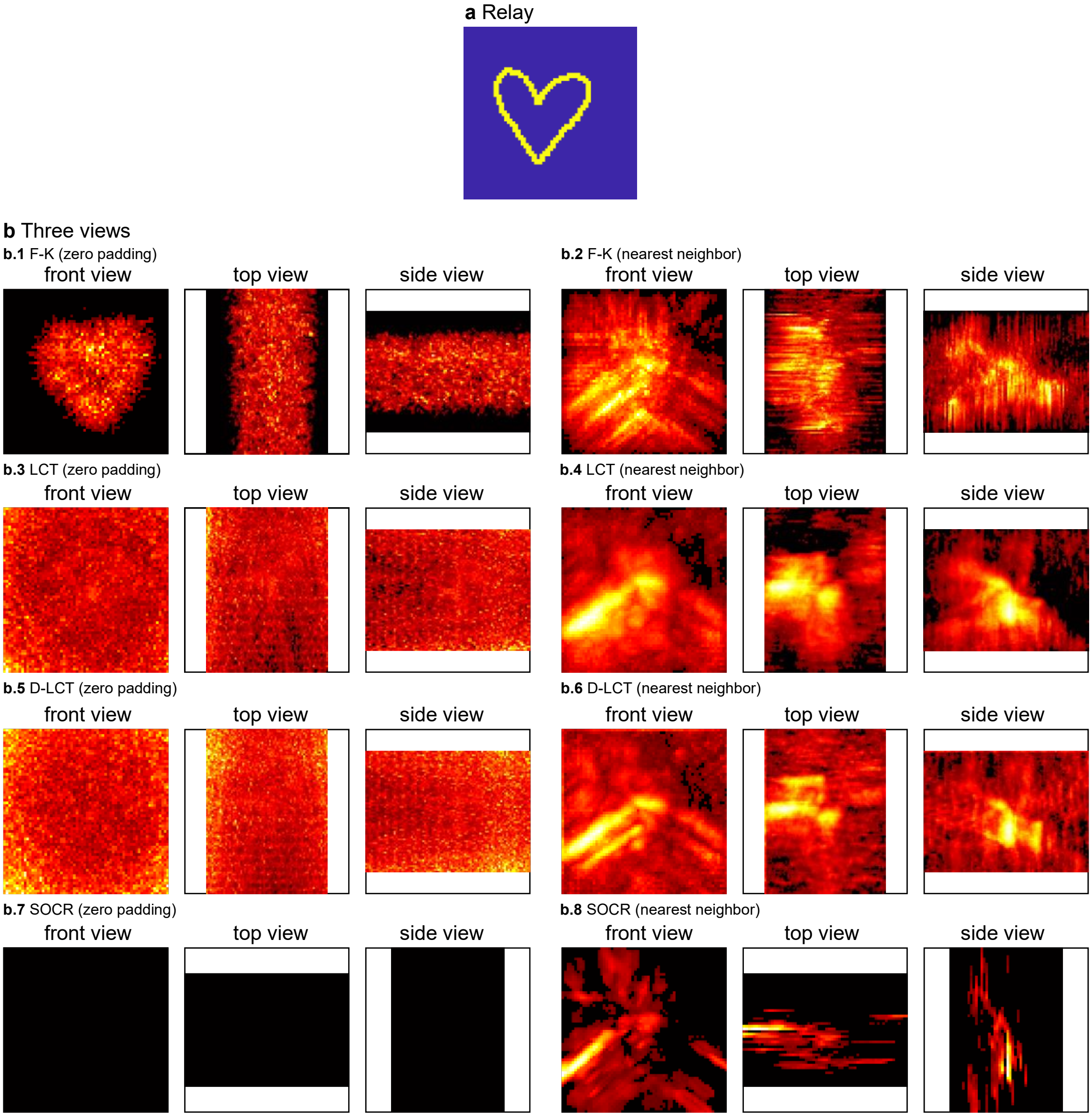

Supplementary Figure 24 Reconstructions of the statue with a heart-shaped relay surface. The signal is preprocessed with two techniques: zero padding and the nearest neighbor interpolation. **a** The relay is a heart-shaped region, which contains 258 focal points. **b** Three views of the F-K, LCT, D-LCT and SOCR reconstructions. For the F-K, LCT, and D-LCT methods, the length of the voxels in the depth direction is 0.48 cm. For the SOCR method, the length of voxels in the depth direction is 0.96 cm. These methods fail to reconstruct the target (See also Supplementary Figure 17).

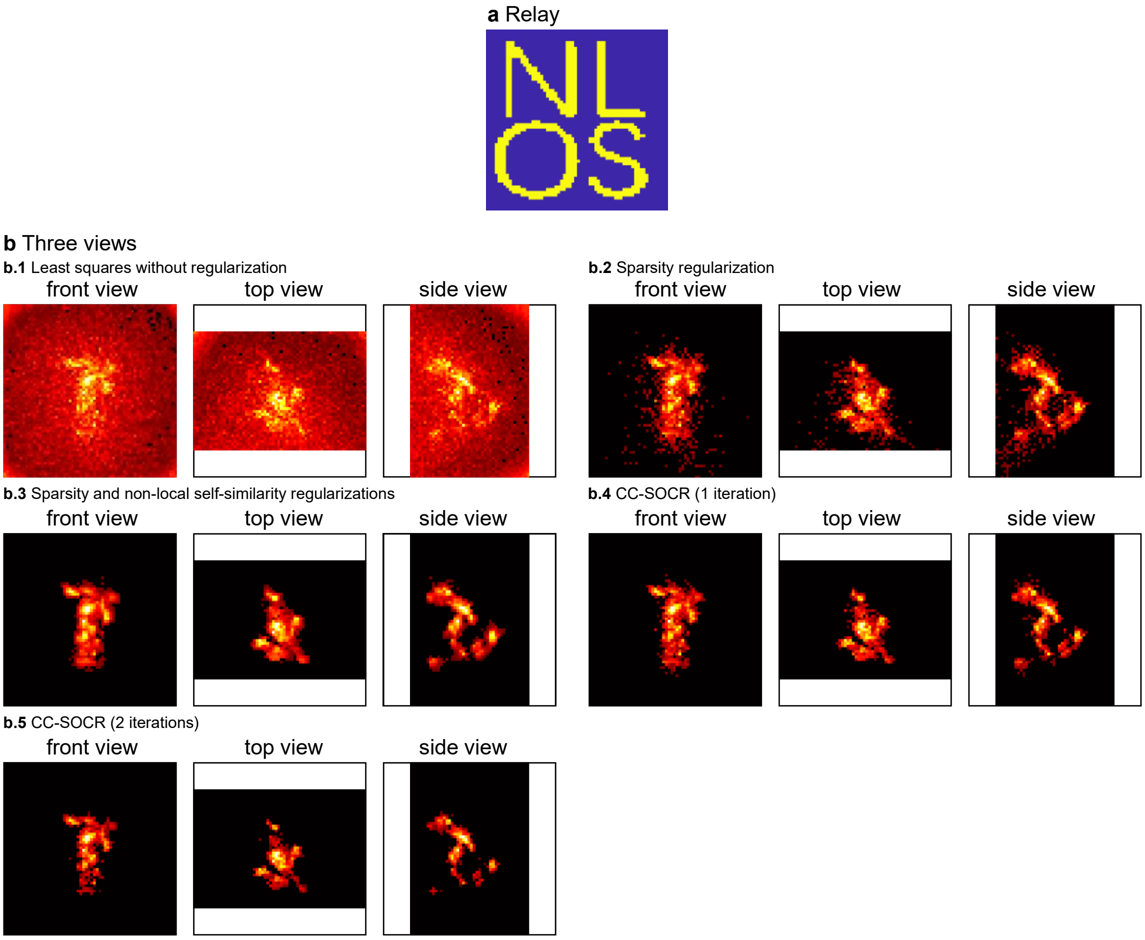

Supplementary Figure 25 Reconstructions of the statue with confocal signals measured at the letters N, L, O and S under different regularization settings. a The relay is the region consisting of four letters N, L, O and S, which includes 825 focal points. **b** Three views of the reconstructions. The least-squares reconstruction without regularizations is of poor quality. The sparsity and non-local self-similarity priors improve the reconstruction quality. The CC-SOCR method reconstructs the target faithfully.

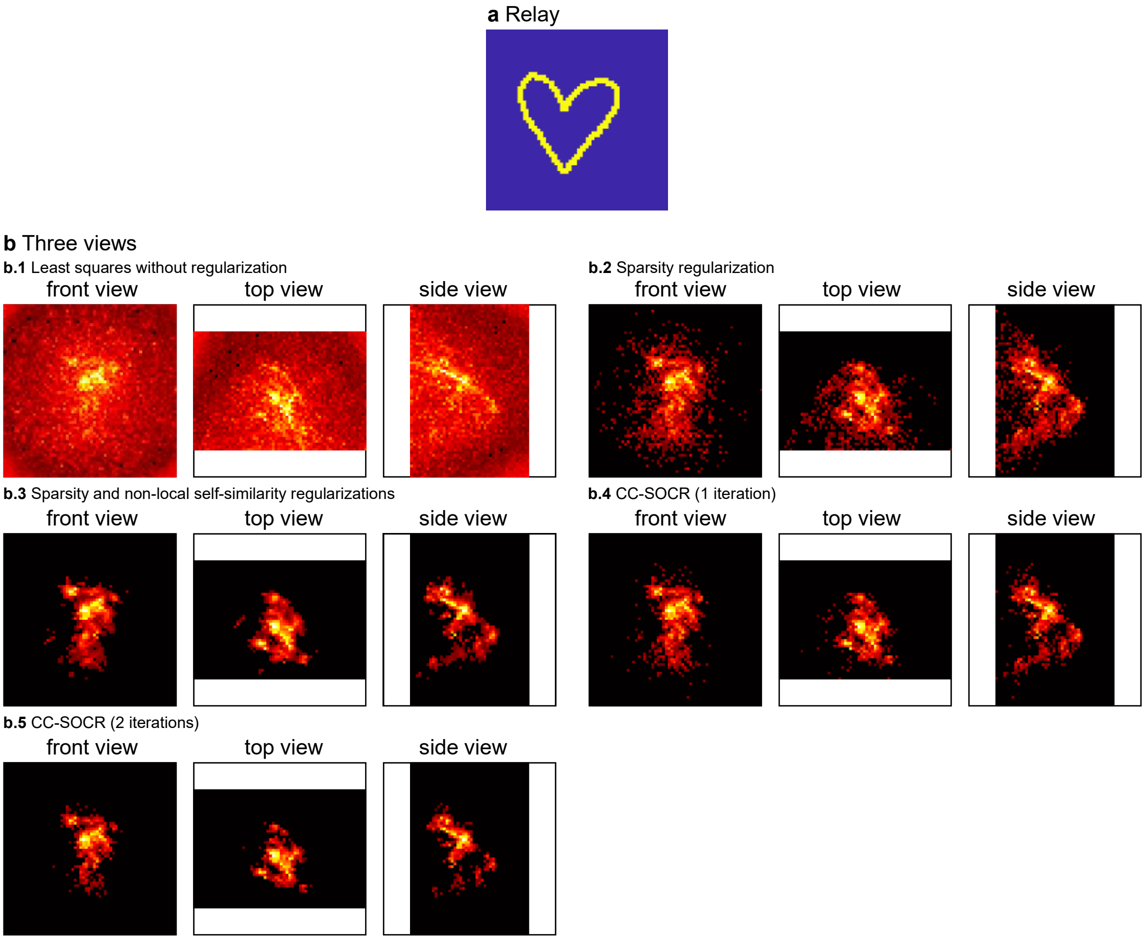

Supplementary Figure 26 Reconstructions of the statue with a heart-shaped relay surface under different regularization settings. a The relay is a heart-shaped region, which contains 258 focal points. **b** Three views of the reconstructions. The least-squares reconstruction without regularizations is of poor quality. The sparsity and non-local self-similarity priors improve the reconstruction quality. The CC-SOCR method reconstructs the target faithfully.

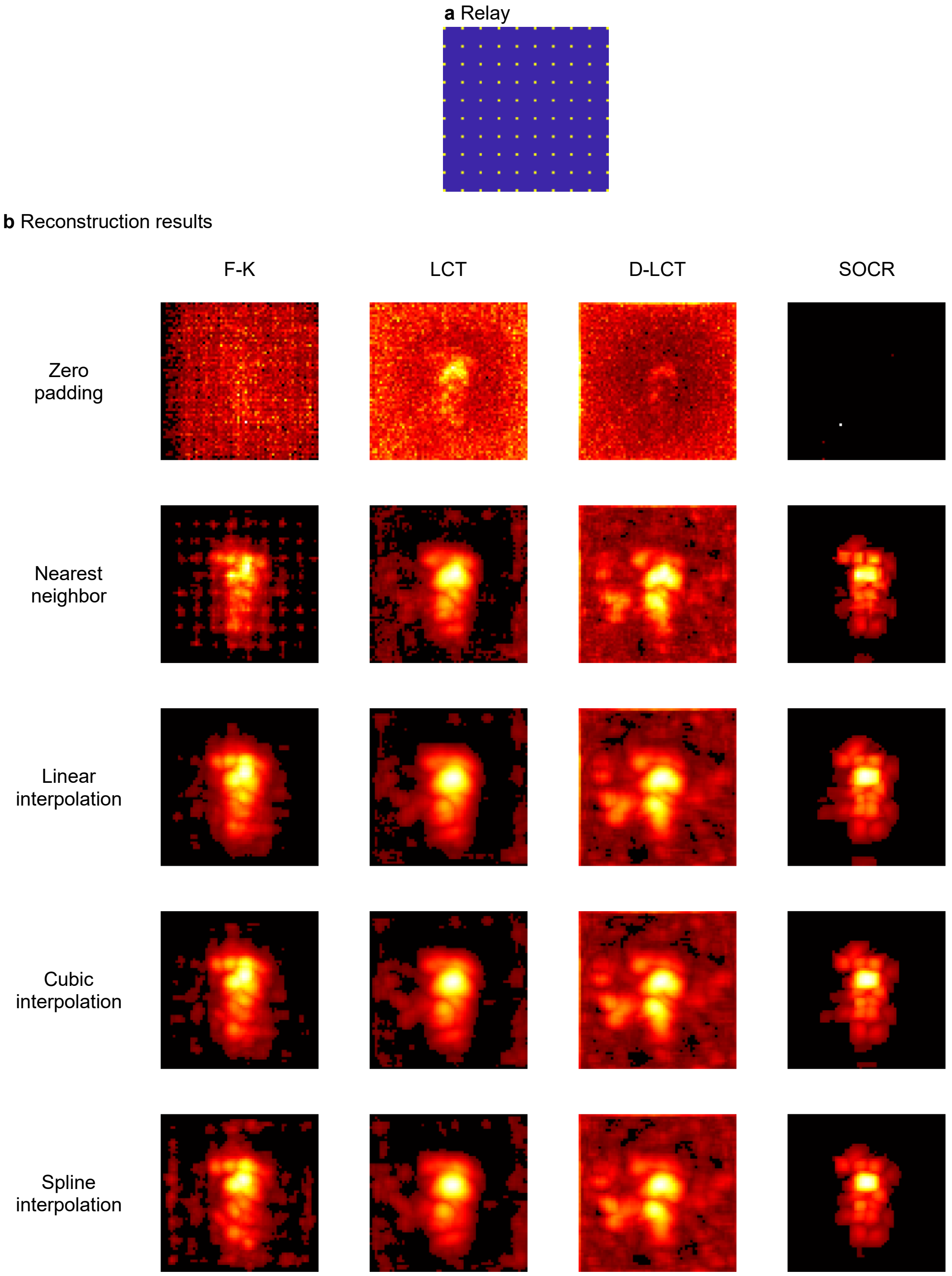

Supplementary Figure 27 Reconstructions of the statue with 10 × 10 focal points and different signal interpolation techniques. a Confocal signals are measured at 10 × 10 focal points. **b** The front view of the reconstructed albedo of the F-K, LCT, D-LCT and SOCR methods. The target cannot be clearly reconstructed with traditional signal interpolation techniques and conventional NLOS imaging algorithms.

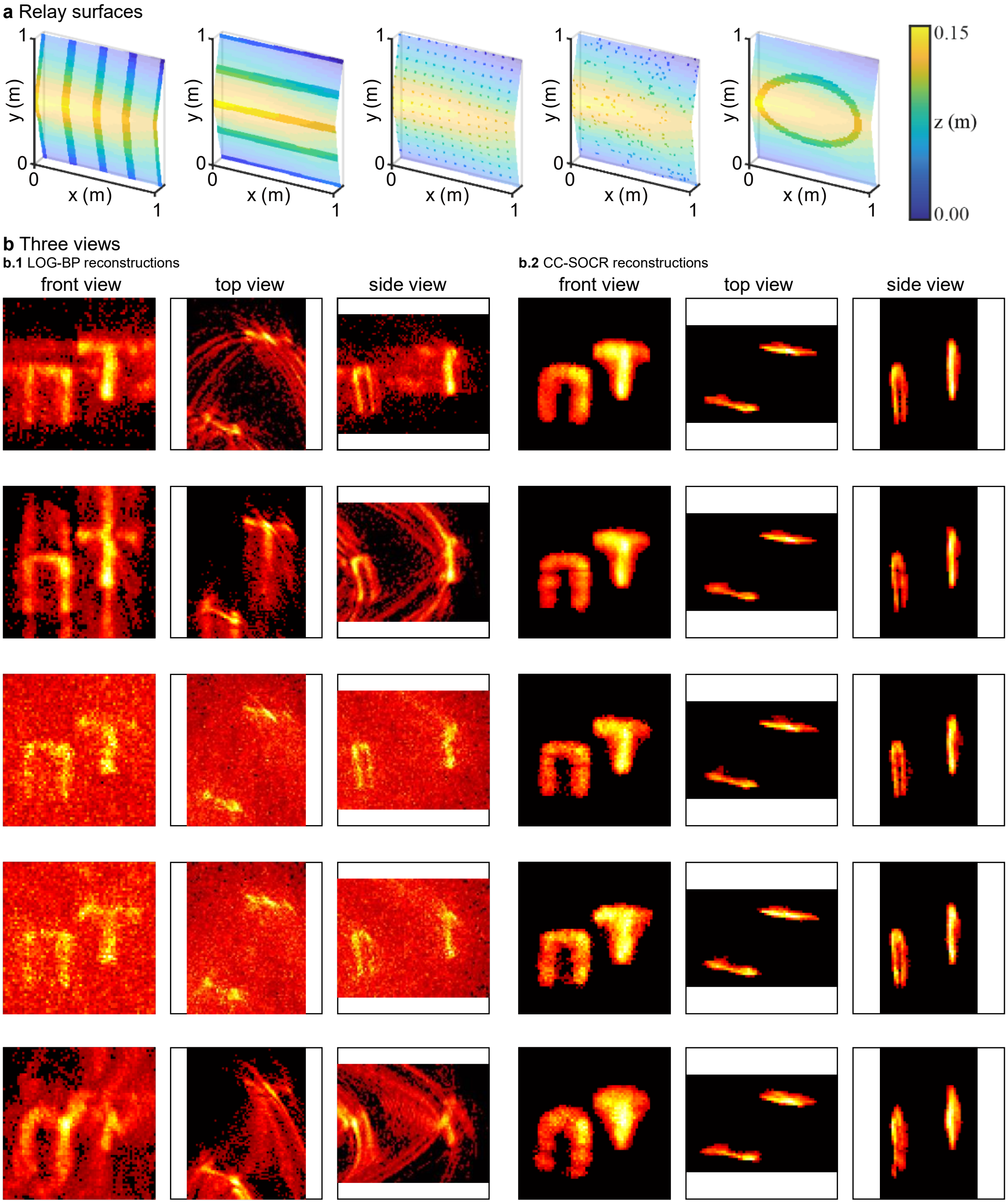

Supplementary Figure 28 Reconstructions of the letters N and T with non-planar relay settings. **a** The illumination regions are shown in opaque. **b** The LOG-BP and CC-SOCR methods directly work under these relay settings. The three views of the reconstruction results with vertical, horizontal, sparse, random, and oval-shaped relay regions are shown from the first to the fifth rows.

Supplementary Note 2 The CC-SOCR algorithm

2.1 Notation

Scalars are represented with lowercase letters. Vectors or matrices are represented with uppercase letters. Uppercase letters in bold are used to represent higher-order tensors. Let a be a positive integer. We use $\mathbb{R}[a]$ to denote the Euclidean space of dimension a. The identity matrix of order a is denoted by $I[a]$. For a tensor $\mathbf{A}$, we denote by $|\mathbf{A}|_0$ the number of non-zero elements of $\mathbf{A}$. We also denote by $\|\mathbf{A}\|_1$ the summation of the absolute values of elements of $\mathbf{A}$. The reconstruction domain is discretized with $n_x \times n_y \times n_z$ voxels. Let $\mathbf{L} \in \mathbb{R}[n_x \times n_y \times n_z]$ and $\mathbf{n} \in \mathbb{R}[n_x \times n_y \times n_z \times 3]$ be the albedo and unit surface normal of the target. The directional albedo $\mathbf{u} \in \mathbb{R}[n_x \times n_y \times n_z \times 3]$ is defined as $\mathbf{u}(i_1, i_2, i_3, j) = \mathbf{L}(i_1, i_2, i_3)\mathbf{n}(i_1, i_2, i_3, j)$ for all $i_1 = 1, \ldots, n_x$, $i_2 = 1, \ldots, n_y$, $i_3 = 1, \ldots, n_z$ and $j = 1, 2, 3$. Noting that the vector field $\mathbf{n}$ has unit length at each voxel, the albedo is given by

$$\mathbf{L}(i_1, i_2, i_3) = \sqrt{\sum_{j=1}^{3} \mathbf{u}(i_1, i_2, i_3, j)^2} \tag{S.1}$$

In the following, we use $\mathbf{L} = \mathrm{albedo}(\mathbf{u})$ as a shorthand of equation (S.1). We also denote $\|\mathbf{L}\|_1$ as $\|\mathbf{u}\|_{2,1}$. Let $\tilde{\mathbf{b}} \in \mathbb{R}[M \times T]$ be the signal measured at M pairs of illumination and detection points on the relay surface, in which T is the maximum number of time bins used. The approximated signal and virtual confocal signal are denoted by $\mathbf{b} \in \mathbb{R}[M \times T]$ and $\mathbf{d} \in \mathbb{R}[n_x n_y \times T]$.

2.2 The CC-SOCR algorithm

The proposed CC-SOCR optimization problem for NLOS reconstruction writes

$$
\begin{aligned}
\min_{\mathbf{u},\mathbf{b},\mathbf{d},D_S,D_n,\mathbf{C},\mathbf{S},\Psi,\mathbf{Q}} & \|A_{\mathbf{b}}\mathbf{u}-\mathbf{b}\|^2 + s_u\|\mathbf{L}\|_1 + s_b|\mathbf{b}|_0 \\
& + \lambda_u \sum_i [\|B_i(\mathbf{L}) - D_s C_i D_n^T\|^2 + \lambda_{pu}|C_i|_0] \\
& + \lambda_b \left\|\mathbf{b}-\tilde{\mathbf{b}}\right\|^2 + \lambda_b\lambda_{pb}\sum_i \left\|P_i(\tilde{\mathbf{b}}) - DS_i\right\|^2 \\
& + \lambda_b\lambda_{pb}\sum_{i,j}\left[\frac{\sigma_{\mathbf{b}}}{d_j^T P_i(A_{\mathbf{b}}\mathbf{u})} S_i(j)\right]^2 \\
& + \lambda_b\lambda_{pb}\lambda_{sb}\sum_i \|P_i(\mathbf{b}) - DS_i\|^2 \\
& + \lambda_d\|A_{\mathbf{d}}\mathbf{u}-\mathbf{d}\|^2 + s_d|\mathbf{d}|_0 \\
& + \lambda_d\lambda_{pd}\sum_i\|Q_i - \Psi^T P_i(\mathbf{d})\|^2 \\
& + \lambda_d\lambda_{pd}\lambda_{sd}\sum_i\|Q_i - \Psi^T P_i(A_{\mathbf{d}}\mathbf{u})\|^2 \\
& + \lambda_d\lambda_{pd}\lambda_{fd}\sum_i |Q_i|_0 \\
& + \lambda_{bd}\|R_{\mathbf{b}}(\mathbf{b},\mathbf{d}) - R_{\mathbf{d}}(\mathbf{b},\mathbf{d})\|^2 \\
\text{s.t.} \quad & \mathbf{L} = \text{albedo}(\mathbf{u}), \\
& D_s^T D_s = I[p_x p_y p_z],\ D_n^T D_n = I[r], \\
& \Psi^T\Psi = I[q_x q_y q_t]
\end{aligned}
\tag{S.2}
$$

in which i is the index of a local patch, P_i is the patch generating operator, B_i is the block matching operator. D represents the matrix of the discrete cosine transform, with its jth filter denoted by d_j. $S_i(j)$ represents the jth element of the vector S_i. $\mathbf{C}$, $\mathbf{S}$ and $\mathbf{Q}$ represent the collections of the transform-domain coefficients $\{C_i\}$, $\{S_i\}$ and $\{Q_i\}$ respectively. $R_{\mathbf{b}}(\mathbf{b},\mathbf{d})$ and $R_{\mathbf{d}}(\mathbf{b},\mathbf{d})$ are the subsets of the approximated signals $\mathbf{b}$ and $\mathbf{d}$ that share the same measurement pairs. p_x, p_y and p_z are the sizes of the local patches of the albedo. r is the maximum number of neighbors kept in the block matching process. q_x, q_y and q_t are the patch sizes of the virtual confocal signal in the horizontal, vertical and temporal directions. s_u, s_b, s_d, σ_d, λ_u, λ_b, λ_d, λ_{pu}, λ_{pb}, λ_{pd}, λ_{sb}, λ_{sd}, λ_{fd} and λ_{bd} are fixed parameters.

In Supplementary Algorithm 1 we present an iterative scheme to solve the NLOS reconstruction problem (S.2). The only input is the measured signal $\tilde{\mathbf{b}}$. The algorithm contains three stages. In the first stage, all variables are initialized by solving the sub-problems. In the second stage, the approximated signal $\mathbf{b}$, the reconstructed target $\mathbf{u}$ and the virtual confocal signal $\mathbf{d}$ are updated sequentially. In the third stage, the albedo and surface normal are computed directly from $\mathbf{u}$. Solutions to the sub-problems (1.2), (1.3), (1.4), (2.2), (2.4) and (2.6) have been studied in the supplementary information of the work of the SOCR method[5]. In the following, we provide a detailed discussion of the remaining sub-problems. We begin with the definition of the hard-thresholding operator. For a real number a and a positive real number y, the hard-thresholding operator $\mathcal{T}$ is

given by

$$\mathcal{T}(a,y)=\begin{cases} 0, & |a|<y \\ a, & |a|\geq y \end{cases} \tag{S.3}$$

For a tensor $\mathbf{A}$, this operator is applied elementwise. With this definition, the solution to the sub-problem (1.1) is expressed as

$$\mathbf{b}^0=\mathcal{T}\left(\tilde{\mathbf{b}},\sqrt{\frac{s_b}{\lambda_b}}\right) \tag{S.4}$$

For the sub-problem (1.5), if the jth measurement pair of the virtual confocal signal does not appear in the measured signal, the solution is given by

$$\mathbf{d}^0[j]=\mathcal{T}\left(A_{\mathbf{d}}\mathbf{u}^0[j],\ \sqrt{\frac{s_d}{\lambda_d}}\right) \tag{S.5}$$

in which $A_{\mathbf{d}}\mathbf{u}^0[j]$ is the simulated signal of the jth measurement pair. Otherwise, the solution writes

$$\mathbf{d}^0[j]=\mathcal{T}\left(\frac{\lambda_d A_{\mathbf{d}}\mathbf{u}^0[j]+\lambda_{bd}\mathbf{b}_j^0}{\lambda_d+\lambda_{bd}},\ \sqrt{\frac{s_d}{\lambda_d+\lambda_{bd}}}\right) \tag{S.6}$$

in which $A_{\mathbf{d}}\mathbf{u}^0[j]$ and $\mathbf{b}_j^0$ are the simulated signal of the jth measurement pair and the corresponding signal of $\mathbf{b}^0$, respectively.

The sub-problem (1.6) is related to frequency domain sparse approximation with orthonormal dictionary atoms[9]. Note that the objective function can be written equivalently as

$$J_1(\Psi,\mathbf{Q})=(1+\lambda_{sd})\sum_i\left[\left\|Q_i-\frac{\Psi^T P_i(\mathbf{d}^0+\lambda_{sd}A_{\mathbf{d}}\mathbf{u}^0)}{1+\lambda_{sd}}\right\|^2+\frac{\lambda_{fd}}{1+\lambda_{sd}}|Q_i|_0\right] \tag{S.7}$$

in which Ψ is an orthogonal matrix. In order to solve this problem with convergence guarantee, it suffices to generalize the data-driven tight frame image denoising algorithm[9] to three dimensions and apply it to $(\mathbf{d}^0+\lambda_{sd}A_{\mathbf{d}}\mathbf{u}^0)/(1+\lambda_{sd})$ with the regularization parameter $\lambda_{fd}/(1+\lambda_{sd})$.

For the sub-problem (2.1), if the jth measurement pair of the measured signal does not appear in the virtual confocal signal, the solution is given by

$$\mathbf{b}^{k+1}[j]=\mathcal{T}\left(\frac{A_{\mathbf{b}}\mathbf{u}^k[j]+\lambda_b\tilde{\mathbf{b}}[j]+\lambda_b\lambda_{pb}\lambda_{sb}P^*(D\mathbf{S}^k)[j]}{\lambda_{b,pb,sb}},\ \sqrt{\frac{s_b}{\lambda_{b,pb,sb}}}\right) \tag{S.8}$$

in which $\lambda_{b,pb,sb}=1+\lambda_b+\lambda_b\lambda_{pb}\lambda_{sb}$. Otherwise,

$$\mathbf{b}^{k+1}[j]=\mathcal{T}\left(\frac{A_{\mathbf{b}}\mathbf{u}^k[j]+\lambda_b\tilde{\mathbf{b}}[j]+\lambda_b\lambda_{pb}\lambda_{sb}P^*(D\mathbf{S}^k)[j]+\lambda_{bd}\mathbf{d}_j^k}{\lambda_{b,pb,sb,bd}},\ \sqrt{\frac{s_b}{\lambda_{b,pb,sb,bd}}}\right) \tag{S.9}$$

in which $\lambda_{b,pb,sb,bd}=1+\lambda_b+\lambda_b\lambda_{pb}\lambda_{sb}+\lambda_{bd}$. $A_{\mathbf{b}}\mathbf{u}^k$ and $\mathbf{d}_j^k$ are the simulated signal of the jth measurement pair and the corresponding signal of $\mathbf{d}^k$, respectively. P^* represents the operator that aggregates the patches back to the signal. $\mathbf{S}$ is the collection

of the Wiener coefficients in the frequency domain. $D\mathbf{S}^k$ is an abbreviation of $\{DS_i^k\}_{i\in I}$, where I is the set of indices of the patches.

The reconstructed target is updated by solving the sub-problem (2.3). In this sub-problem, the term $\phi(\mathbf{u})=\sum_{i,j}\left(\frac{\sigma_{\mathbf{b}}}{d_j^T P_i(A_{\mathbf{b}}\mathbf{u})}S_i^{k+1}(j)\right)^2$ is omitted. Otherwise, the problem will be non-linear and difficult to solve. This problem contains a L_1-regularization term, which can be solved efficiently with the split Bregman method[10]. Convergence is guaranteed if the sub-problems are solved accurately. The scheme is shown in Supplementary Algorithm 2, in which the sub-problem of updating $\mathbf{v}_{j+1}^{k+1}$ has a closed-form solution as follows

$$\mathbf{v}_{j+1}^{k+1}(i_1,i_2,i_3,h)=\max\left(0,1-\frac{s_u}{2\mu\|\mathbf{u}_j^{k+1}(i_1,i_2,i_3,h)-\mathbf{q}_j^{k+1}(i_1,i_2,i_3,h)\|}\right)\cdot(\mathbf{u}_j^{k+1}(i_1,i_2,i_3,h)-\mathbf{q}_j^{k+1}(i_1,i_2,i_3,h)) \tag{S.10}$$

in which i_1, i_2 and i_3 are indices of the voxel in three directions and $h=1,2,3$. To update $\mathbf{u}_{j+1}^{k+1}$, we minimize the following objective function

$$\begin{aligned}J_2(\mathbf{u})=&\|A_{\mathbf{b}}\mathbf{u}-\mathbf{b}^{k+1}\|^2\\&+(\lambda_d+\lambda_d\lambda_{pd}\lambda_{sd})\left\|A_{\mathbf{d}}\mathbf{u}-\frac{\lambda_d\mathbf{d}^k+\lambda_d\lambda_{pd}\lambda_{sd}P^*(\Psi^k\mathbf{Q}^k)}{\lambda_d+\lambda_d\lambda_{pd}\lambda_{sd}}\right\|^2\\&+(\lambda_u+\mu)\left\|\mathbf{u}-\frac{\lambda_u B^*\left(D_s^k\mathbf{C}^k(D_n^k)^T\right)+\mu(\mathbf{v}_{j+1}^{k+1}+\mathbf{q}_j^{k+1})}{\lambda_u+\mu}\right\|^2\end{aligned} \tag{S.11}$$

in which $\mathbf{Q}^k$ and $\mathbf{C}^k$ are the collections of transform-domain coefficients, P^* and B^* are the operators that aggregate the patch dataset or block dataset back to the signal and albedo. $\Psi^k\mathbf{Q}^k$ and $D_s^k\mathbf{C}^k(D_n^k)^T$ are understood as $\{\Psi^k Q_i^k|i\in I\}$ and $\{D_s^k C_i^k(D_n^k)^T|i\in I\}$. Noting that $B^*\left(D_s^k\mathbf{C}^k(D_n^k)^T\right)$ is a three-dimensional volume that does not contain information of the surface normal, we use the technique introduced in SOCR[5] to construct a directional albedo with the surface normal provided by $\mathbf{u}^k$. See the supplement of SOCR[5] for more details. Here, we abuse the notation and also use $B^*\left(D_s^k\mathbf{C}^k(D_n^k)^T\right)$ to represent this directional albedo. Minimizing the objective function (S.11) yields a least-squares problem without constraint, which can be solved with the conjugate gradient method.

We remark that this sub-problem is solved approximately due to the omitted term $\phi(\mathbf{u})$ and the treatment of $B^*\left(D_s^k\mathbf{C}^k(D_n^k)^T\right)$. Nonetheless, extensive experimental results in Supplementary Note 1 indicate that high-quality reconstructions can be obtained with these tricks.

For the sub-problem (2.5), if the jth measurement pair of the virtual confocal signal does not appear in the measured signal, we have

$$\mathbf{d}^{k+1}[j]=\mathcal{T}\left(\frac{A_{\mathbf{d}}\mathbf{u}^{k+1}[j]+\lambda_{pd}P^*(\Psi^k\mathbf{Q}^k)[j]}{1+\lambda_{pd}},\sqrt{\frac{s_d}{\lambda_d+\lambda_d\lambda_{pd}}}\right) \tag{S.12}$$

Otherwise, the solution writes

$$\mathbf{d}^{k+1}[j]=\mathcal{T}\left(\frac{\lambda_d A_{\mathbf{d}}\mathbf{u}^{k+1}[j]+\lambda_d\lambda_{pd}P^*(\Psi^k\mathbf{Q}^k)[j]+\lambda_{bd}\mathbf{b}_j^{k+1}}{\lambda_d+\lambda_d\lambda_{pd}+\lambda_{bd}},\sqrt{\frac{s_d}{\lambda_d+\lambda_d\lambda_{pd}+\lambda_{bd}}}\right) \tag{S.13}$$

in which $A_{\mathbf{d}}\mathbf{u}^{k+1}[j]$ and $\mathbf{b}_j^{k+1}$ are the simulated signal of the jth measurement pair and the corresponding signal of $\mathbf{b}^{k+1}$, respectively.

The sub-problem (2.6) is of the same type as (1.6) and can be solved using the same method discussed above.

Supplementary Note 3 The choice of parameters

There are 14 parameters in the CC-SOCR optimization problem (S.2), namely s_u, s_b, λ_u, λ_{pu}, λ_b, λ_{pb}, $\sigma_{\mathbf{b}}$, λ_{sb}, λ_d, s_d, λ_{pd}, λ_{sd}, λ_{fd} and λ_{bd}. In this section, we show how these parameters are chosen adaptively.

3.1 Parameters chosen in the initialization stage

The input signal $\tilde{\mathbf{b}}$ is normalized into the range $[0,255]$ in advance. In step (1.1), the approximated signal $\mathbf{b}$ is initialized by applying the hard-thresholding operator to the input signal. The threshold value $\sqrt{s_b/\lambda_b}$ depends on two parameters, and we set $s_b^{imp}=\sqrt{s_b/\lambda_b}=255\times 0.01$. In step (1.2), a sparse reconstruction is initialized. The parameter s_u and an additional parameter μ introduced for the split Bregman iteration are initialized as s_u^{init} and μ^{init} by directly following the work of SOCR[5]. We will adjust these two parameters in step (2.3). In step (1.3), the parameter λ_{pu} can be chosen implicitly following the method presented in the previous work[5]. In step (1.4), we fix $\lambda_{sb}=0.25$. The parameter $\sigma_{\mathbf{b}}$ is related to the noise level of the input signal and is chosen from the set $\{20,40,60\}$. The sub-problem (1.5) involves three parameters λ_d, λ_{bd} and s_d. In this sub-problem, we choose $\lambda_d=2$ (The parameter λ_d will be fixed in step (2.3)). The parameter λ_{bd} is fixed as 4 throughout the reconstruction process. In equations (S.5) and (S.6), we simply set $s_d^{imp}=\sqrt{s_d/\lambda_d}=\sqrt{s_d/(\lambda_d+\lambda_{bd})}=255\times 0.01$ despite $\lambda_{bd}\neq 0$. In step (1.6), we fix $\lambda_{sd}=1$ to assign equal weights to $\mathbf{d^0}$ and $A_{\mathbf{d}}\mathbf{u}^0$ in the process of dictionary learning. The parameter λ_{fd} is implicitly chosen such that the corresponding virtual noise level belongs to the set $\{20,40,60\}$[5,9].

3.2 Parameters determined in the first iteration

In the first iteration, all parameters are fixed. In step (2.1), noting that the hard-thresholding operator applies to a convex combination of $A_{\mathbf{b}}\mathbf{u}^0$, $\tilde{\mathbf{b}}$, $P^*(D\mathbf{S}^0)$ and $\mathbf{d}^0$ with weights $1:\lambda_b:\lambda_b\lambda_{pb}\lambda_{sb}:\lambda_{bd}$, we fix $\lambda_b=1$, $\lambda_{pb}=16$ and $\lambda_{bd}=4$. The parameter s_b is then determined as $s_b=\lambda_b\left(s_b^{imp}\right)^2$. In step (2.3), the reconstructed target is updated. By setting the terms

$$\|A_{\mathbf{b}}\mathbf{u}^0-\mathbf{b}^1\|^2 : \left\|\mathbf{u}^0-B^*\left(D_s^0\mathbf{C}^0(D_n^0)^T\right)\right\|^2 : \|A_{\mathbf{d}}\mathbf{u}^0-\mathbf{d}^0\|^2 = 1:\lambda_u^{imp}:\lambda_b^{imp} \tag{S.14}$$

The parameters λ_u and λ_d are adaptively chosen as

$$\lambda_u=\lambda_u^{imp}\frac{\|A_{\mathbf{b}}\mathbf{u}^0-\mathbf{b}^1\|^2}{\left\|\mathbf{u}^0-B^*\left(D_s^0\mathbf{C}^0(D_n^0)^T\right)\right\|^2} \tag{S.15}$$

and

$$\lambda_d=\lambda_d^{imp}\frac{\|A_{\mathbf{b}}\mathbf{u}^0-\mathbf{b}^1\|^2}{\|A_{\mathbf{d}}\mathbf{u}^0-\mathbf{d}^0\|^2} \tag{S.16}$$

In all experiments, we set $\lambda_u^{imp} \in [0, 50]$ and fix $\lambda_d^{imp} = 2$. We also fix $\lambda_{pd} = 4$ to assign a large weight to the contribution of $P^*(\Psi^0 \mathbf{Q}^0)$. Besides, comparing sub-problems (1.2) and (2.3), we fix s_u and μ as

$$s_u = s_u^{init}(1 + \lambda_u^{imp} + \lambda_d^{imp}(1 + \lambda_{pd} + \lambda_{sd})) \tag{S.17}$$

$$\mu = \mu^{init}(1 + \lambda_u^{imp} + \lambda_d^{imp}(1 + \lambda_{pd} + \lambda_{sd})) \tag{S.18}$$

The virtual confocal signal is updated in step (2.5). By setting the truncation value in equation (S.12) as $s_d^{imp} = \sqrt{s_d / (\lambda_d + \lambda_d \lambda_{pd})} = 255 \times 0.01$, we fix s_d as

$$s_d = (\mathrm{s}_d^{imp})^2 \lambda_d (1 + \lambda_{pd}) \tag{S.19}$$

3.3 Remarks

Remark 1: For the instance of the pyramid, the parameters s_b^{imp} and s_d^{imp} are set as 255×0.001 because the generated simulated signal does not contain background noise.

Remark 2: In steps (1.3) and (2.4), the patch sizes and the searching window sizes for block matching and dictionary learning need manual tuning. Interested readers are referred to existing works[5,9,11] for the choices of these parameters.

Remark 3: In steps (1.4) and (2.2), the patch size of the signal is fixed as 1 × 1 × 3. In steps (1.6) and (2.6), the patch size of the signal is fixed as 3 × 3 × 3.

Remark 4: For all experiments, the final results are obtained in no more than 2 iterations. In steps (1.2) and (2.3), the conjugate gradient (CG) method is used to obtain the least-squares solutions. The maximum number of CG iterations is set to 20 and the iterations stop whenever the relative error of the normal equation reaches below 0.005. In these two sub-problems, the maximum numbers of the split Bregman iterations (Supplementary Algorithm 2) are fixed as 20 and 3 respectively. These iterations stop whenever the relative errors of the updated variables $\mathbf{u}$ and $\mathbf{v}$ reach below 0.005.

Supplementary Note 4 Time and memory complexity

Consider a typical setting where the reconstruction domain is discretized with $N \times N \times N$ voxels and the signal is detected at M measurement pairs. When the virtual confocal signals are considered at $N \times N \times N$ focal points and $p_x,\ p_y,\ p_z,\ q_x,\ q_y,\ T,\ s,\ r,\ w = \mathcal{O}(1)$ (See Supplementary Table 1 for the meaning of these parameters), the time and memory complexity of the CC-SOCR method are $\mathcal{O}(\max\{N^5, MN^3\})$ and $\mathcal{O}(\max\{N^3, MN\})$, respectively. Comparisons of the time and memory complexity with other methods are provided in Supplementary Table 2.

4.1 Time complexity

The overall time complexity of sub-problems (1.2), (1.3), (1.4), (2.2) and (2.4) is $\mathcal{O}(\max\{N^5, MN^3\})$, as discussed in section 4 of the supplement of the work[5]. For the rest of the sub-problems, it takes $\mathcal{O}(MN)$ to apply the elementwise hard-thresholding in step (1.1). In step (1.5), it takes $\mathcal{O}(N^5)$ to generate the simulated signal $A_d\mathbf{u}^0$ and $\mathcal{O}(N^3)$ to initialize $\mathbf{d}$ according to equations (S.5) and (S.6). By writing the objective function of sub-problem (1.6) equivalently as equation (S.7), we conclude that the time complexity of this step is no more than that of step (1.3), because it does not contain the block matching process. In sub-problem (2.1), generating the simulated signal costs $\mathcal{O}(MN^3)$, and it takes $\mathcal{O}(MN)$ to update $\mathbf{b}$ with equations (S.8) and (S.9). The sub-problem (2.3) is solved with Supplementary Algorithm 2, which takes $\mathcal{O}(\max\{N^5, MN^3\})$ to solve the least-squares problem and $\mathcal{O}(N^3)$ to update $\mathbf{v}_{k+1}^{j+1}$ and $\mathbf{q}_{k+1}^{j+1}$. The time complexity of step (2.5) is the same as that of step (1.5), which requires $\mathcal{O}(N^5)$. The sub-problem (2.6) is exactly of the same type as the sub-problem (1.6). To sum up, the overall time complexity of the CC-SOCR algorithm is $\mathcal{O}(\max\{N^5, MN^3\})$.

4.2 Memory complexity

To store the input signal $\tilde{\mathbf{b}}$, $\mathcal{O}(MN)$ memory is needed. It takes $\mathcal{O}(N^3)$ to store the physical linear operator due to the repetition of elements in the measurement matrices $A_\mathbf{b}$ and $A_\mathbf{d}$. Generating the simulated signal with the target takes $\mathcal{O}(\max\{MN, N^3\})$ memory with the linear operator-based implementation of the physical model. The steps (1.1), (1.5), (2.1) and (2.5) take $\mathcal{O}(\max\{MN, N^3\})$ memory because the solutions are obtained with pointwise truncation. The sub-problems (1.2) and (2.3) also take $\mathcal{O}(\max\{MN, N^3\})$ storage, where solutions of the linear systems are obtained with the conjugate gradient method. For the sub-problems (1.3) and (2.4), it suffices to compute $B^*(D_s\mathbf{C}D_n^T)$, which can be implemented block by block with $\mathcal{O}(N^3)$ memory[11]. In steps (1.4) and (2.2), it suffices to compute $P^*(D\mathbf{S})$, which can be realized using the sliding window Wiener filtering technique with $\mathcal{O}(N^3)$ storage[11]. For the sub-problems (1.6) and (2.6), it suffices to compute $P^*(\Psi\mathbf{Q})$, which takes $\mathcal{O}(N^3)$ memory with a patch by patch implementation of the data-driven tight frame denoising algorithm[9]. In all, the memory complexity of the proposed method is $\mathcal{O}(\max\{MN, N^3\})$.

4.3 Execution time

The execution time of the CC-SOCR algorithm for the instance of the statue with 200 randomly distributed confocal measurements and virtual confocal signals of different sizes are shown in Supplementary Tables 3 - 6. The code was run on an AMD EPYC 7452 server with 64 cores. It is shown that sparser virtual confocal signals result in shorter execution time. However, the reconstruction quality decreases with the size of the virtual confocal signal (See Supplementary Figure 23).

Supplementary Algorithm 1 Solving the CC-SOCR optimization problem

Stage 1: Initialization

(1.1) Initialize the approximated signal.

$$\mathbf{b}^0 = \underset{\mathbf{b}}{\operatorname{argmin}}\ \lambda_b \left\| \mathbf{b} - \tilde{\mathbf{b}} \right\|^2 + s_b |\mathbf{b}|_0$$

(1.2) Initialize the reconstructed target.

$$\mathbf{u}^0 = \underset{\mathbf{u}}{\operatorname{argmin}}\ \| A_{\mathbf{b}} \mathbf{u} - \mathbf{b}^0 \|^2 + s_u \| \mathbf{u} \|_{2,1}$$

(1.3) Initialize the dictionaries of the albedo.

$$(D_s^0, D_n^0, \mathbf{C}^0) = \underset{D_s, D_n, \mathbf{C}}{\operatorname{argmin}}\ \sum_i \left(\| B_i(\mathbf{L}^0) - D_s C_i D_n^T \|^2 + \lambda_{pu} |C_i|_0 \right)$$
$$\text{s.t.}\quad \mathbf{L}^0 = \text{albedo}(\mathbf{u}^0),\quad D_s^T D_s = I[p_x p_y p_z],\quad D_n^T D_n = I[r]$$

(1.4) Initialize the Wiener coefficients.

$$\mathbf{S}^0 = \underset{\mathbf{S}}{\operatorname{argmin}}\ \sum_i \left[\left\| P_i(\tilde{\mathbf{b}}) - DS_i \right\|^2 + \lambda_{sb} \| P_i(\mathbf{b}^0) - DS_i \|^2 + \sum_j \left(\frac{\sigma_{\mathbf{b}}}{d_j^T P_i(A_{\mathbf{b}} \mathbf{u}^0)} S_i(j) \right)^2 \right]$$

(1.5) Initialize the virtual confocal signal.

$$\mathbf{d}^0 = \underset{\mathbf{d}}{\operatorname{argmin}}\ \lambda_d \| A_{\mathbf{d}} \mathbf{u}^0 - \mathbf{d} \|^2 + \lambda_{bd} \| R_{\mathbf{b}^0}(\mathbf{b}^0, \mathbf{d}) - R_{\mathbf{d}}(\mathbf{b}^0, \mathbf{d}) \|^2 + s_d |\mathbf{d}|_0$$

(1.6) Initialize the dictionary of the virtual confocal signal.

$$(\Psi^0, \mathbf{Q}^0) = \underset{\Psi, \mathbf{Q}}{\operatorname{argmin}}\ \sum_i \left(\| Q_i - \Psi^T P_i(\mathbf{d}^0) \|^2 + \lambda_{sd} \| Q_i - \Psi^T P_i(A_{\mathbf{d}} \mathbf{u}^0) \|^2 + \lambda_{fd} |Q_i|_0 \right)$$
$$\text{s.t.}\quad \Psi^T \Psi = I[q_x q_y q_t]$$

Stage 2: Iteration

For $k = 0, 1, \ldots, K-1$

(2.1) Update the approximated signal.

$$\mathbf{b}^{k+1} = \underset{\mathbf{b}}{\operatorname{argmin}}\ \| A_{\mathbf{b}} \mathbf{u}^k - \mathbf{b} \|^2 + \lambda_b \left\| \mathbf{b} - \tilde{\mathbf{b}} \right\|^2 + s_b |\mathbf{b}|_0$$
$$+ \lambda_b \lambda_{pb} \lambda_{sb} \| P_i(\mathbf{b}) - D(S_i)^k \|^2 + \lambda_{bd} \| R_{\mathbf{b}}(\mathbf{b}, \mathbf{d}^k) - R_{\mathbf{d}^k}(\mathbf{b}, \mathbf{d}^k) \|^2$$

(2.2) Update the Wiener coefficients.

$$\mathbf{S}^{k+1} = \underset{\mathbf{S}}{\operatorname{argmin}}\ \sum_i \left[\left\| P_i(\tilde{\mathbf{b}}) - DS_i \right\|^2 + \lambda_{sb} \| P_i(\mathbf{b}^{k+1}) - DS_i \|^2 + \sum_j \left(\frac{\sigma_{\mathbf{b}}}{d_j^T P_i(A_{\mathbf{b}} \mathbf{u}^k)} S_i(j) \right)^2 \right]$$

(2.3) Update the reconstructed target.

$$\mathbf{u}^{k+1} = \underset{\mathbf{u}}{\operatorname{argmin}}\ \| A_{\mathbf{b}} \mathbf{u} - \mathbf{b}^{k+1} \|^2 + \lambda_u \sum_i \| B_i(\mathbf{L}) - D_s^k C_i^k (D_n^k)^T \|^2 + s_u \| \mathbf{u} \|_{2,1}$$
$$+ \lambda_d \| A_{\mathbf{d}} \mathbf{u} - \mathbf{d}^k \|^2 + \lambda_d \lambda_{pd} \lambda_{sd} \sum_i \| Q_i^k - (\Psi^k)^T P_i(A_{\mathbf{d}} \mathbf{u}) \|^2$$
$$\text{s.t.}\quad \mathbf{L} = \text{albedo}(\mathbf{u})$$

(2.4) Update the dictionaries of the albedo.

$$(D_s^{k+1}, D_n^{k+1}, \mathbf{C}^{k+1}) = \underset{D_s, D_n, \mathbf{C}}{\operatorname{argmin}} \sum_i \left(\| B_i(\mathbf{L}^{k+1}) - D_s C_i D_n^T \|^2 + \lambda_{pu} |C_i|_0 \right)$$

$$\text{s.t.} \quad \mathbf{L}^{k+1} = \operatorname{albedo}(\mathbf{u}^{k+1}), \quad D_s^T D_s = I[p_x p_y p_z], \quad D_n^T D_n = I[r]$$

(2.5) Update the virtual confocal signal.

$$\mathbf{d}^{k+1} = \underset{\mathbf{d}}{\operatorname{argmin}} \ \lambda_d \| A_{\mathbf{d}} \mathbf{u}^{k+1} - \mathbf{d} \|^2 + \lambda_d \lambda_{pd} \sum_i \| Q_i^k - (\Psi^k)^T P_i(\mathbf{d}) \|^2$$
$$+ \lambda_{bd} \| R_{\mathbf{b}^{k+1}}(\mathbf{b}^{k+1}, \mathbf{d}) - R_{\mathbf{d}}(\mathbf{b}^{k+1}, \mathbf{d}) \|^2 + s_d |\mathbf{d}|_0$$

(2.6) Update the dictionary of the virtual confocal signal.

$$(\Psi^{k+1}, \mathbf{Q}^{k+1}) = \underset{\Psi, \mathbf{Q}}{\operatorname{argmin}} \sum_i \left(\| Q_i - \Psi^T P_i(\mathbf{d}^{k+1}) \|^2 + \lambda_{sd} \| Q_i - \Psi^T P_i(A_{\mathbf{d}} \mathbf{u}^{k+1}) \|^2 + \lambda_{fd} |Q_i|_0 \right)$$

$$\text{s.t.} \quad \Psi^T \Psi = I[q_x q_y q_t]$$

End

Stage 3: Output results

(3.1) The reconstructed albedo is given by $\mathbf{L}^* = \operatorname{albedo}(\mathbf{u}^K)$.

(3.2) The reconstructed surface normal is given by $\mathbf{n}^* = \mathbf{u}^K / \mathbf{L}^*$.

Supplementary Algorithm 2 Updating the reconstructed target

$\mathbf{q}_0^{k+1} = \mathbf{0}$

$\mathbf{u}_0^{k+1} = \mathbf{u}^k$

For $j = 0, 1, \ldots, J-1$

$\mathbf{v}_{j+1}^{k+1} = \underset{\mathbf{v}}{\operatorname{argmin}}\ s_u \| \mathbf{v} \|_{2,1} + \mu \| \mathbf{v} - \mathbf{u}_j^{k+1} + \mathbf{q}_j^{k+1} \|^2$

$$\mathbf{u}_{j+1}^{k+1} = \underset{\mathbf{u}}{\operatorname{argmin}} \| A_{\mathbf{b}} \mathbf{u} - \mathbf{b}^{k+1} \|^2 + \lambda_u \sum_i \| B_i(\mathbf{L}) - D_s^k C_i^k (D_n^k)^T \|^2 + \lambda_d \| A_{\mathbf{d}} \mathbf{u} - \mathbf{d}^k \|^2$$

$$+ \lambda_d \lambda_{pd} \lambda_{sd} \sum_i \| Q_i^k - (\Psi^k)^T P_i(A_{\mathbf{d}} \mathbf{u}) \|^2 + \mu \| \mathbf{v}_{j+1}^{k+1} - \mathbf{u} + \mathbf{q}_j^{k+1} \|^2$$

$$\text{s.t.} \quad \mathbf{L} = \text{albedo}(\mathbf{u})$$

$\mathbf{q}_{j+1}^{k+1} = \mathbf{q}_j^{k+1} + \mathbf{v}_{j+1}^{k+1} - \mathbf{u}_{j+1}^{k+1}$

End

$\mathbf{u}^{k+1} = \mathbf{v}_J^{k+1}$

Supplementary Table 1 Parameters of the experimental setup

Parameter	Explanation
n_x, n_y, n_z	The number of voxels in the horizontal, vertical and depth directions
p_x, p_y, p_z	The patch sizes of the albedo in the horizontal, vertical and depth directions
q_x, q_y, q_t	The patch sizes of the virtual signal in the horizontal, vertical and temporal directions
r	The number of neighboring blocks of each local albedo block
w	Searching window size of the albedo block in the process of block matching
M	The number of measurement pairs
s	The patch size of the measured signal in the temporal direction
T	The number of time bins for each measurement pair

Supplementary Table 2 Comparisons of the time and memory complexity of NLOS imaging algorithms with M measurement pairs and N × N × N voxels

Method	Time complexity	Memory complexity
LOG-BP[8]	$\mathcal{O}(MN^3)$	$\mathcal{O}(N^3)$
LCT[2]	$\mathcal{O}(N^3 \log N)$	$\mathcal{O}(N^3)$
D-LCT[3]	$\mathcal{O}(N^3 \log N)$	$\mathcal{O}(N^3)$
F-K[1]	$\mathcal{O}(N^3 \log N)$	$\mathcal{O}(N^3)$
PF-RSD[4]	$\mathcal{O}(N^3 \log N)$	$\mathcal{O}(N^3)$
SOCR[5]	$\mathcal{O}(N^5)$	$\mathcal{O}(N^3)$
CC-SOCR	$\mathcal{O}(\max\{MN^3, N^5\})$	$\mathcal{O}(\max\{MN, N^3\})$

Supplementary Table 3 Execution time of the instance of the statue with 200 randomly distributed confocal measurements and 64 × 64 virtual confocal signal

Sub-problem	Explanation	CPU time (s)	
Computing the forward operator of the physical model		0.1937	
**Stage 1: Initialization**			
(1.1)	Initializing **b**	0.0017	
(1.2)	Initializing **u**	60.9705	
(1.3)	Initializing D_s, D_n and **C**	7.0017	
(1.4)	Initializing **S**	0.8245	
(1.5)	Initializing **d**	1.9342	
(1.6)	Initializing Ψ and **Q**	10.8064	
**Stage 2: Iteration**			
(2.1)	Updating **b**	0.0267	0.7261
(2.2)	Updating **S**	0.0333	0.0165
(2.3)	Updating **u**	82.4961	81.7546
(2.4)	Updating D_s, D_n and **C**	4.8466	4.4856
(2.5)	Updating **d**	2.0045	2.0358
(2.6)	Updating Ψ and **Q**	10.6700	10.5273
**Total execution time (s)**			
281.3559			

Supplementary Table 4 Execution time of the instance of the statue with 200 randomly distributed confocal measurements and 32 × 32 virtual confocal signal

Sub-problem	Explanation	CPU time (s)	
Computing the forward operator of the physical model		0.1892	
**Stage 1: Initialization**			
(1.1)	Initializing $\mathbf{b}$	0.0018	
(1.2)	Initializing $\mathbf{u}$	63.3050	
(1.3)	Initializing D_s, D_n and $\mathbf{C}$	7.4353	
(1.4)	Initializing $\mathbf{S}$	0.8742	
(1.5)	Initializing $\mathbf{d}$	1.2871	
(1.6)	Initializing Ψ and $\mathbf{Q}$	3.5379	
**Stage 2: Iteration**			
(2.1)	Updating $\mathbf{b}$	0.0165	0.8005
(2.2)	Updating $\mathbf{S}$	0.0378	0.0134
(2.3)	Updating $\mathbf{u}$	40.3780	44.9235
(2.4)	Updating D_s, D_n and $\mathbf{C}$	4.5323	4.6030
(2.5)	Updating $\mathbf{d}$	1.2301	1.1797
(2.6)	Updating Ψ and $\mathbf{Q}$	3.3823	3.3888
**Total execution time (s)**			
181.1166			

Supplementary Table 5 Execution time of the instance of the statue with 200 randomly distributed confocal measurements and 16 × 16 virtual confocal signal

Sub-problem	Explanation	CPU time (s)	
Computing the forward operator of the physical model		0.1918	
**Stage 1: Initialization**			
(1.1)	Initializing $\mathbf{b}$	0.0015	
(1.2)	Initializing $\mathbf{u}$	67.0203	
(1.3)	Initializing D_s, D_n and $\mathbf{C}$	7.4327	
(1.4)	Initializing $\mathbf{S}$	0.8427	
(1.5)	Initializing $\mathbf{d}$	0.8562	
(1.6)	Initializing Ψ and $\mathbf{Q}$	1.1980	
**Stage 2: Iteration**			
(2.1)	Updating $\mathbf{b}$	0.0216	0.9710
(2.2)	Updating $\mathbf{S}$	0.0361	0.0184
(2.3)	Updating $\mathbf{u}$	34.5548	39.0236
(2.4)	Updating D_s, D_n and $\mathbf{C}$	4.4882	3.8257
(2.5)	Updating $\mathbf{d}$	0.7508	0.7671
(2.6)	Updating Ψ and $\mathbf{Q}$	1.1771	1.1989
**Total execution time (s)**			
164.3765			

Supplementary Table 6 Execution time of the instance of the statue with 200 randomly distributed confocal measurements and 8 × 8 virtual confocal signal

Sub-problem	Explanation	CPU time (s)	
Computing the forward operator of the physical model		0.2316	
**Stage 1: Initialization**			
(1.1)	Initializing $\mathbf{b}$	0.0016	
(1.2)	Initializing $\mathbf{u}$	67.0207	
(1.3)	Initializing D_s, D_n and $\mathbf{C}$	7.3978	
(1.4)	Initializing $\mathbf{S}$	0.9241	
(1.5)	Initializing $\mathbf{d}$	0.5542	
(1.6)	Initializing Ψ and $\mathbf{Q}$	0.5237	
**Stage 2: Iteration**			
(2.1)	Updating $\mathbf{b}$	0.0068	0.8318
(2.2)	Updating $\mathbf{S}$	0.0470	0.0149
(2.3)	Updating $\mathbf{u}$	23.6866	28.4683
(2.4)	Updating D_s, D_n and $\mathbf{C}$	4.5322	4.4342
(2.5)	Updating $\mathbf{d}$	0.5168	0.4928
(2.6)	Updating Ψ and $\mathbf{Q}$	0.4959	0.4342
**Total execution time (s)**			
140.6152			

Supplementary References

1. Lindell, D. B., Wetzstein, G. & O'Toole, M. Wave-based non-line-of-sight imaging using fast *f-k* migration. *ACM Trans. Graph.* **38**, 1–13 (2019).
2. Matthew O'Toole, Lindell, D. B. & Wetzstein, G. Confocal non-line-of-sight imaging based on the light-cone transform. *Nature* **555**, 338–341 (2018).
3. Young, S. I., Lindell, D. B., Girod, B., Taubman, D. & Wetzstein, G. Non-Line-of-Sight Surface Reconstruction Using the Directional Light-Cone Transform. in *2020 IEEE/CVF Conference on Computer Vision and Pattern Recognition (CVPR)* 1404–1413 (IEEE, 2020). doi:10.1109/CVPR42600.2020.00148.
4. Liu, X., Bauer, S. & Velten, A. Phasor field diffraction based reconstruction for fast non-line-of-sight imaging systems. *Nat. Commun.* **11**, 1645 (2020).
5. Liu, X. *et al.* Non-line-of-sight reconstruction with signal–object collaborative regularization. *Light Sci. Appl.* **10**, 198 (2021).
6. Galindo, M., Marco, J., O'Toole, M., Wetzstein, G. & Jarabo, A. A dataset for benchmarking time-resolved non-line-of-sight imaging. in *ACM SIGGRAPH 2019 Posters* (2019).
7. Liu, X. *et al.* Non-line-of-sight imaging using phasor-field virtual wave optics. *Nature* **572**, 620–623 (2019).
8. Laurenzis, M. & Velten, A. Feature selection and back-projection algorithms for nonline-of-sight laser–gated viewing. *J. Electron. Imaging* **23**, 063003 (2014).
9. Cai, J.-F., Ji, H., Shen, Z. & Ye, G.-B. Data-driven tight frame construction and image denoising. *Appl. Comput. Harmon. Anal.* **37**, 89–105 (2014).
10. Goldstein, T. & Osher, S. The Split Bregman Method for L1-Regularized Problems. *SIAM J. Imaging*

Sci. **2**, 323–343 (2009).

11. D Abov, K., Foi, A., Katkovnik, V. & Egiazarian, K. Image denoising with block-matching and 3D filtering. in *Proceedings of SPIE - The International Society for Optical Engineering* 354–365 (2006).
