# Supplementary material for: Non-line-of-sight imaging with arbitrary illumination and detection pattern
Source: Nat Commun. 2023 Jun 3;14:3230. doi: 10.1038/s41467-023-38898-4 (PMC10239523; doi:10.1038/s41467-023-38898-4)
Supplement: Supplementary file 3 — Description of Additional Supplementary Files [file 41467_2023_38898_MOESM3_ESM.pdf]

Description of Additional Supplementary Files

Supplementary Software - This is the supplementary code of our work, which contains demos that reproduce the results shown in the manuscript and supplementary datasets cited in the manuscript. The file “demo-code.zip” contains the code that solves the proposed CC-SOCR optimization problem. Details are provided in the file“readme.md”
