# Supplementary figures and images for: Non-line-of-sight imaging with arbitrary illumination and detection pattern
Source: Nat Commun. 2023 Jun 3;14:3230. doi: 10.1038/s41467-023-38898-4 (PMC10239523; doi:10.1038/s41467-023-38898-4)

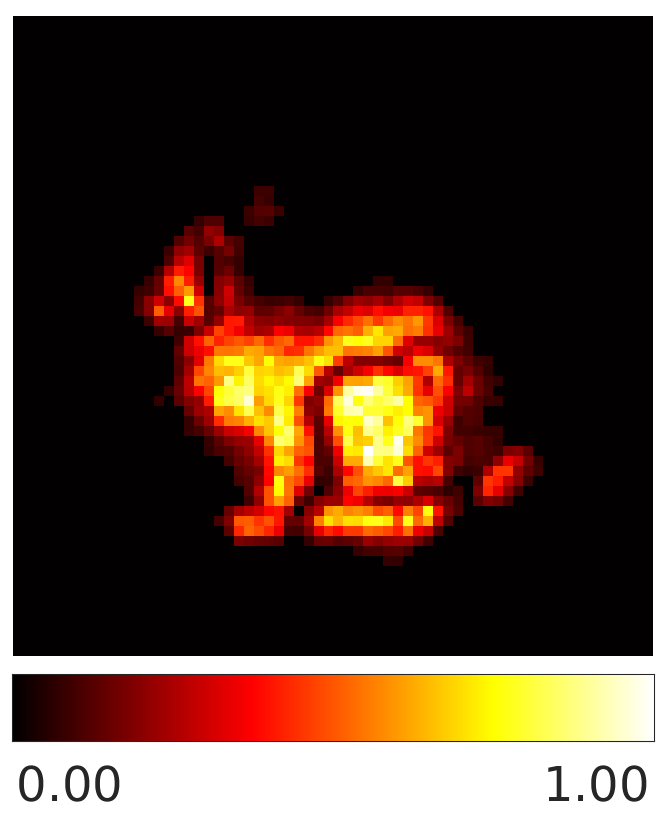

Supplement: Supplementary file 4 — Supplementary Software [file 41467_2023_38898_MOESM4_ESM.zip › demo_code/results_bunny_full/albedo.png]

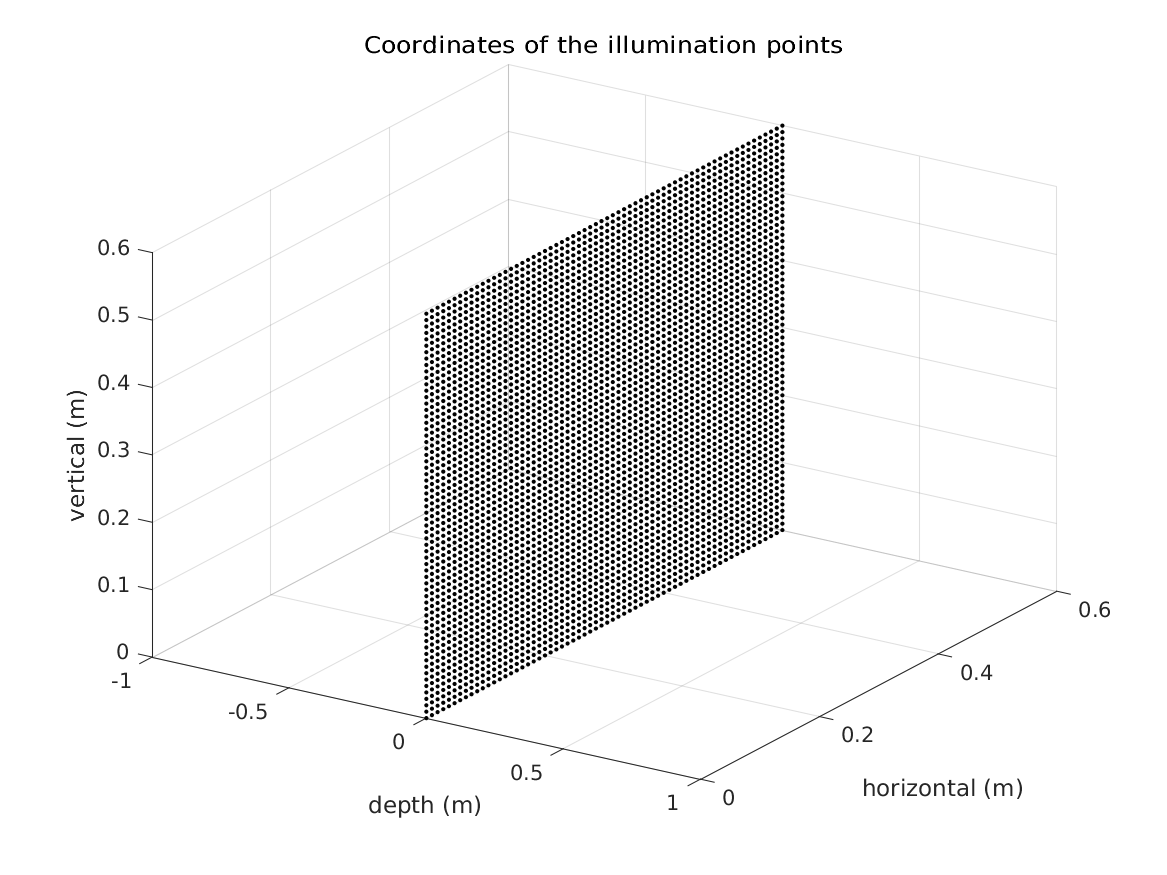

Supplement: Supplementary file 4 — Supplementary Software [file 41467_2023_38898_MOESM4_ESM.zip › demo_code/results_bunny_full/illumination_pattern.png]

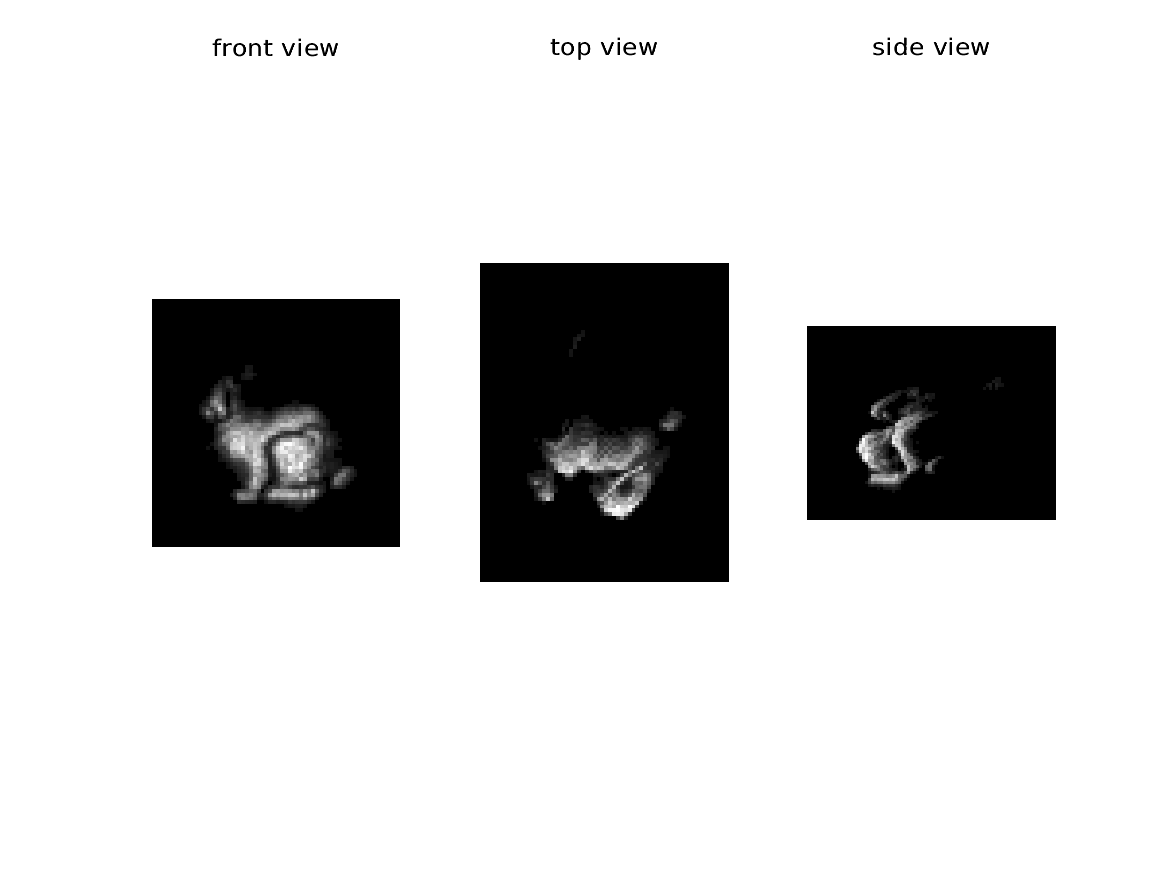

Supplement: Supplementary file 4 — Supplementary Software [file 41467_2023_38898_MOESM4_ESM.zip › demo_code/results_bunny_full/three view.png]

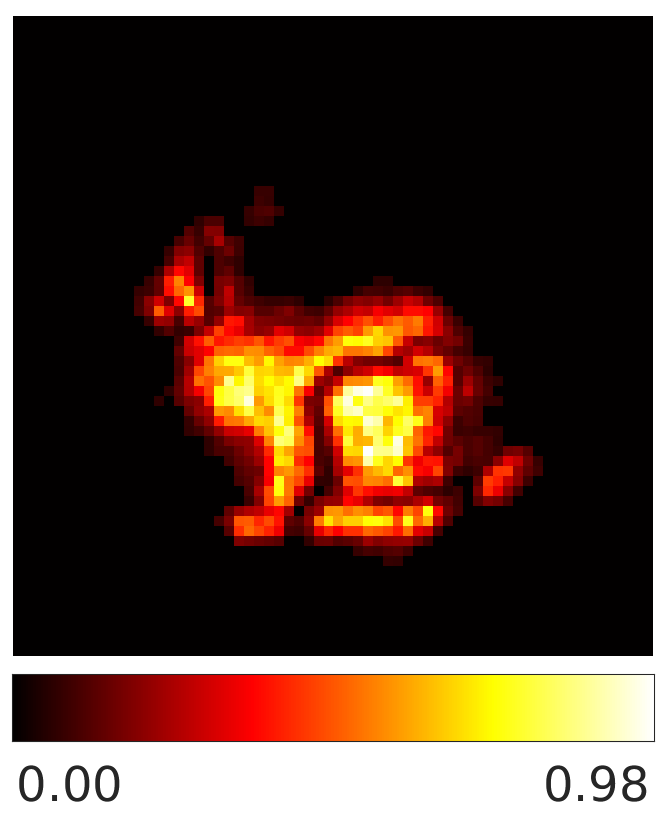

Supplement: Supplementary file 4 — Supplementary Software [file 41467_2023_38898_MOESM4_ESM.zip › demo_code/results_bunny_full/x-component.png]

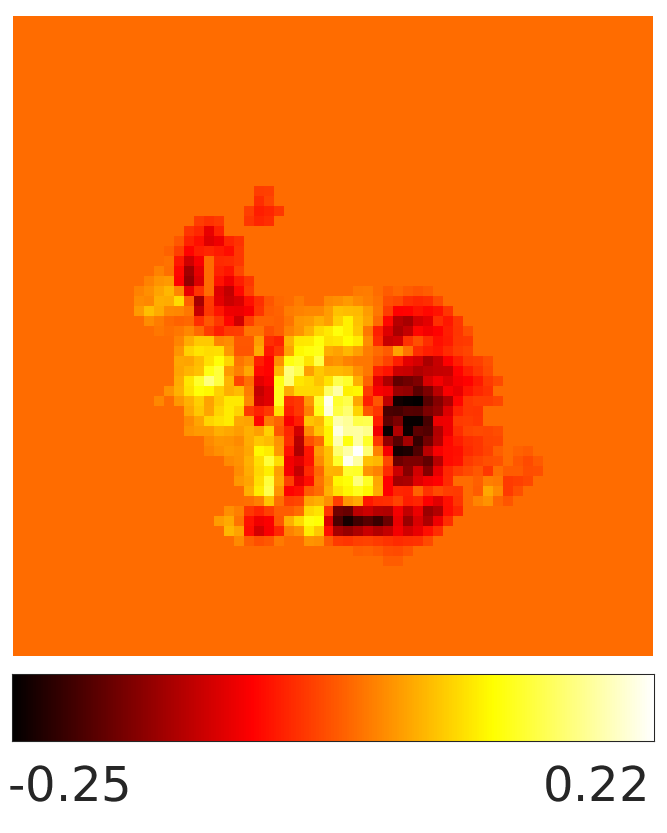

Supplement: Supplementary file 4 — Supplementary Software [file 41467_2023_38898_MOESM4_ESM.zip › demo_code/results_bunny_full/y-component.png]

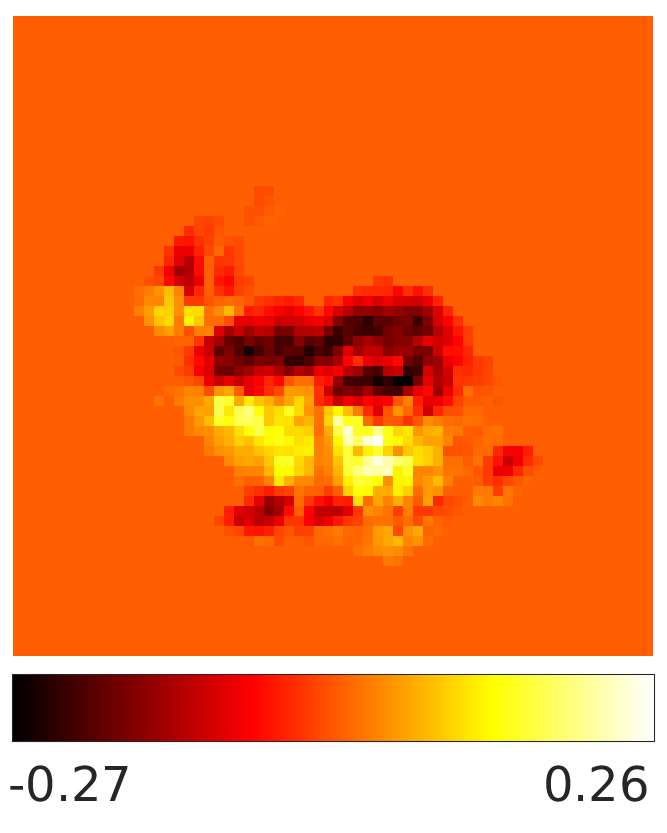

Supplement: Supplementary file 4 — Supplementary Software [file 41467_2023_38898_MOESM4_ESM.zip › demo_code/results_bunny_full/z-component.png]
